# Crystal structure of *E. coli* lipoprotein diacylglyceryl transferase

**DOI:** 10.1038/ncomms10198

**Published:** 2016-01-05

**Authors:** Guotao Mao, Yan Zhao, Xusheng Kang, Zhijie Li, Yan Zhang, Xianping Wang, Fei Sun, Krishnan Sankaran, Xuejun C. Zhang

**Affiliations:** 1 National Laboratory of Macromolecules, National Center of Protein Science - Beijing, Institute of Biophysics, Chinese Academy of Sciences, 15 Datun Road, Beijing 100101, China; 2 University of Chinese Academy of Sciences, Beijing 100049, China; 3 School of Life Sciences, University of Science and Technology of China, Hefei, Anhui 230027, China; 4 Centre for Biotechnology, Anna University, Chennai 600025, India

## Abstract

Lipoprotein biogenesis is essential for bacterial survival. Phosphatidylglycerol:prolipoprotein diacylglyceryl transferase (Lgt) is an integral membrane enzyme that catalyses the first reaction of the three-step post-translational lipid modification. Deletion of the *lgt* gene is lethal to most Gram-negative bacteria. Here we present the crystal structures of *Escherichia coli* Lgt in complex with phosphatidylglycerol and the inhibitor palmitic acid at 1.9 and 1.6 Å resolution, respectively. The structures reveal the presence of two binding sites and support the previously reported structure–function relationships of Lgt. Complementation results of *lgt*-knockout cells with different mutant Lgt variants revealed critical residues, including Arg143 and Arg239, that are essential for diacylglyceryl transfer. Using a GFP-based *in vitro* assay, we correlated the activities of Lgt with structural observations. Together, the structural and biochemical data support a mechanism whereby substrate and product, lipid-modified lipobox-containing peptide, enter and leave the enzyme laterally relative to the lipid bilayer.

## Abstract (web summary)

Bacterial lipoproteins have important biological functions, and the lipoprotien biogenesis enzyme Lgt is essential in most gram-negative bacteria. Here, the authors use structural and biochemical techniques to shed light on the function of Lgt in post-translational transacylation modification.

## Graphical abstract

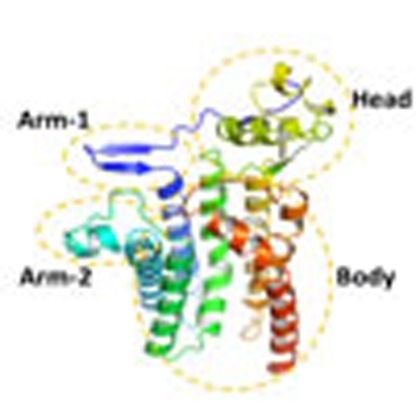

Bacterial lipoproteins fulfill wide ranging and vital biological functions such as maintenance of cell envelope architecture, insertion and stabilization of outer membrane proteins, nutrient uptake, transport, adhesion, invasion and virulence^1^,^2^. Despite their diverse structures, functions and origins, most of these lipoproteins contain *N*-acyldiacylglyceryl-cysteine as their N-terminal amino acid^3^. Similar post-translational modifications of lipoproteins may play important roles in archaea as well^4^ (Supplementary Fig. 1). The biosynthetic pathway for lipoproteins, present both in Gram-negative and Gram-positive bacteria with high GC content, consists of the following three reactions (Fig. 1a): (i) diacylglyceryl modification of pre-prolipoproteins by phosphatidylglycerol:pro*L*ipoprotein diacyl*G*lyceryl *T*ransferase (Lgt) to form diacylglyceryl–prolipoproteins; (ii) cleavage of signal peptide from diacylglyceryl-prolipoproteins by *L*ipoprotein *S*ignal *P*eptidase (LspA) to form apolipoproteins; and (iii) *N*-acylation of apolipoproteins by an apo*L*ipoproteiin *N*-acyl *T*ransferase (Lnt), resulting in mature lipoproteins^1^,^5^. As this pathway has been shown to be essential for survival of Gram-negative bacteria^6^, these enzymes present excellent potential targets for the next generation of broad spectrum antibiotics.

Lgt, which catalyses the first reaction of the pathway, recognizes the ‘lipobox' motif (with the consensus sequence [LVI]^(−3)^[ASTVI]^(−2)^[GAS]^(−1)^C^(+1)^) of prolipoproteins^7^,^8^ and transfers the diacylglyceryl group from phosphatidylglycerol (PG) to the thiol group of the conserved cysteine residue in the lipobox sequence^5^. Functional studies of *Escherichia coli* Lgt (*Ec*Lgt) show that it is encoded by the *lgt* (previously *umpA*) gene^9^ as a 291-amino acid (33 kDa) membrane protein. While mutagenesis studies attempted to identify the amino-acid residues essential for the transacylation activity of Lgt^6^,^10^, the catalytic mechanism of this activity and the structural basis of substrate specificity remained elusive. Here, we report the crystal structures of *Ec*Lgt with lipid ligands at up to 1.6-Å resolution. Our structures revealed a laterally opening central cavity with the active site and two binding sites for PG (the donor substrate). Mutagenesis and *in vitro* studies showed that the key residues essential for transacylation activity are also concentrated in the periplasmic half of the central cavity, and point to a feasible mechanism of substrate recognition.

## Results

### Overall structure–function relationship of recombinant *Ec*Lgt

**Figure 1:**
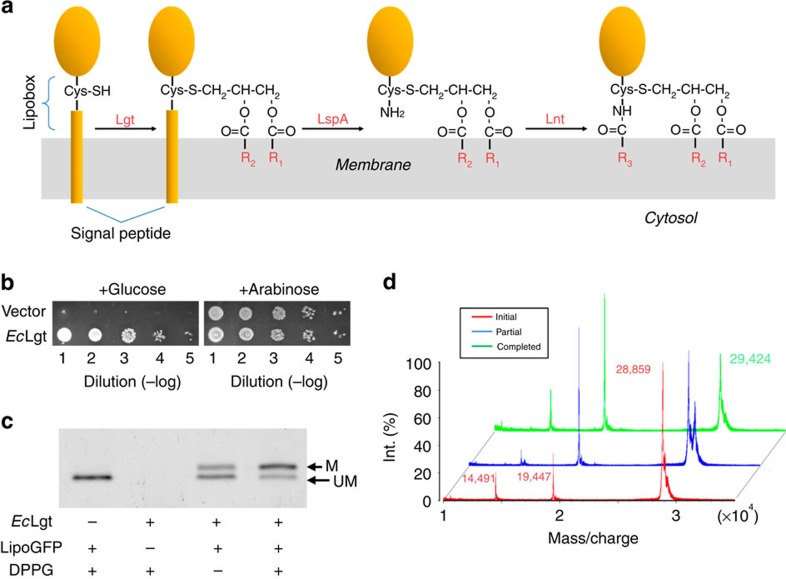
Functional assays of recombinant WT Lgt. (**a**) Schematic diagram of the three-step peptide lipidation reaction. The third reaction only occurs in Gram-negative bacteria. (**b**) Complementation assay of Lgt with the Δ*lgt* cell strain. A serial dilution of cell culture was spotted onto solid medium containing either 11 mM glucose (left columns) or 1 mM arabinose (right columns). Images were taken 16 h after incubation at 37 °C. (**c**) *In vitro* transacylation assay. *Ec*Lgt (1.2 μM) was incubated with lipoGFP (peptide substrate, 7 μM) in the presence or absence of DPPG (donor substrate, 40 μM). An amount of 25 ng of lipoGFP from each reaction was loaded onto the SDS gel. The lower band (UM) is the unmodified lipoGFP (acceptor substrate) and upper band (M) is the lipid-modified lipoGFP (product). Activity in the absence of externally added PG is attributed to the presence of PG associated with Lgt in the purified preparation. Uncropped images are shown in Supplementary Fig. 6. (**d**) MALDI-TOF Mass spectroscopy analysis of the *in vitro* reaction mixtures. During the reaction, the lipoGFP peak moved from 28,859 Da (red peak) to 29,424 Da (green peak), with their mass difference matching well with the calculated value of 559 Da.

Recombinant *Ec*Lgt was overexpressed in *E. coli*. Complementation of *lgt*-conditional depletion strain confirmed the *in vivo* activity of the recombinant *Ec*Lgt (Fig. 1b, also see below). To investigate the *in vitro* activities of recombinant EcLgt, we have developed a gel-mobility assay using a fluorescent lipoGFP as the substrate of *Ec*Lgt. The lipoGFP was engineered with the N-terminal 24 amino-acid residues of major outer membrane lipoprotein Lpp precursor^5^ fused to the N-terminus of GFP. The result clearly demonstrates that the purified *Ec*Lgt does exhibit the diacylglyceryl transferase activity (Fig. 1c,d).

Purified Lgt was initially crystallized in the presence of palmitic acid and the detergent *n*-octyl-β-D-glucoside (OG), both of which were shown to be inhibitors to the Lgt activity (for example, when [OG]>0.5%)^5^,^11^. The crystal structure determined using the Se-methionine-based single wavelength anomalous dispersion phasing method was refined at 1.6-Å resolution (referred to here as form-1). The space group of the crystal form is *P*2_1_2_1_2_1_, and there is one *Ec*Lgt molecule per crystallography asymmetric unit with ca. 55% solvent content (*V*_M_=2.7 Å^3^ Da^−1^), indicating that *Ec*Lgt was present in its monomeric form. The final refined model of *Ec*Lgt contained amino-acid residues 3–286, one palmitic acid molecule, two PG molecules, one detergent (OG) molecule, seven other partial alkyl chains and ca. 100 water molecules. During the screening of Lgt crystals suitable for structure determination, we solved and refined another crystal structure of Lgt, with ligand-binding modes that appeared to differ from form-1 (referred to as form-2). The resolution of this structure was 1.9 Å, and the cell parameters were essentially identical to those of the form-1 crystal. Although the overall structures from the two crystal forms were almost identical (with an r.m.s.d. of 0.52 Å for all Cα atoms), the form-2 structure contained no OG molecule and showed a significant conformational change in the loop L6–7 close to the highly conserved residue R143. More importantly, the form-2 structure contained two additional substrate PG molecules inside the Lgt structure. Statistics of collection of diffraction data and structure refinement are summarized in Table 1.

**Figure 2:**
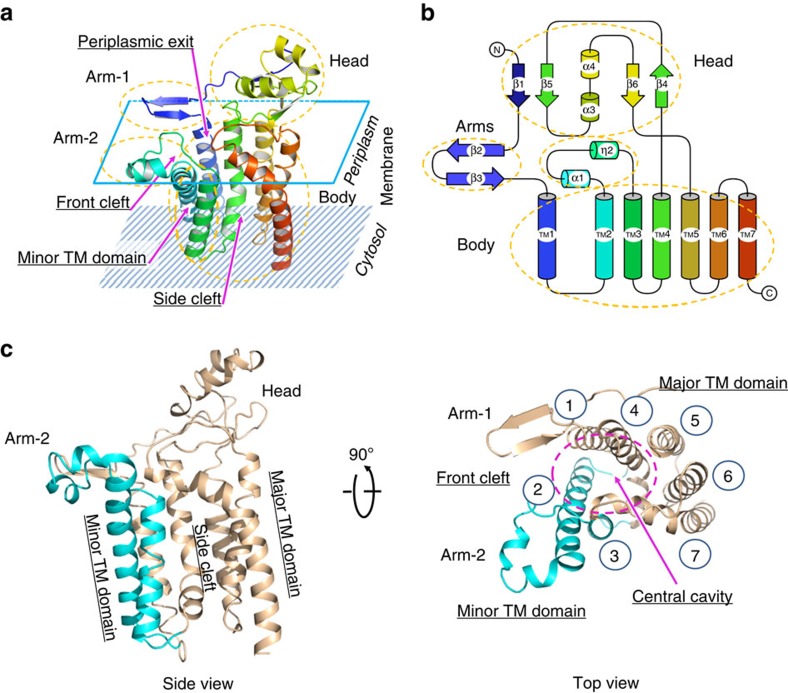
Overall structure of *Ec*Lgt. (**a**) Ribbon presentation. The peptide chain of *Ec*Lgt is coloured in a rainbow scheme, with the N terminus in blue and C terminus in red. Electron densities of the overall structure of *Ec*Lgt are shown in Supplementary Fig. 7. (**b**) Topology of the peptide folding. The seven TM helices (labelled as TM1–TM7) and periplasmic helices (α1, η2, α3 and α4) are shown as cylinders, and β-strands (β1–β6) with arrows. (**c**) Side and top views. The minor TM domain is shown in cyan, and the remaining parts in wheat colour. In the top view, the head domain is removed for clarity, and TM helices are numbered 1–7.

**Figure 3:**
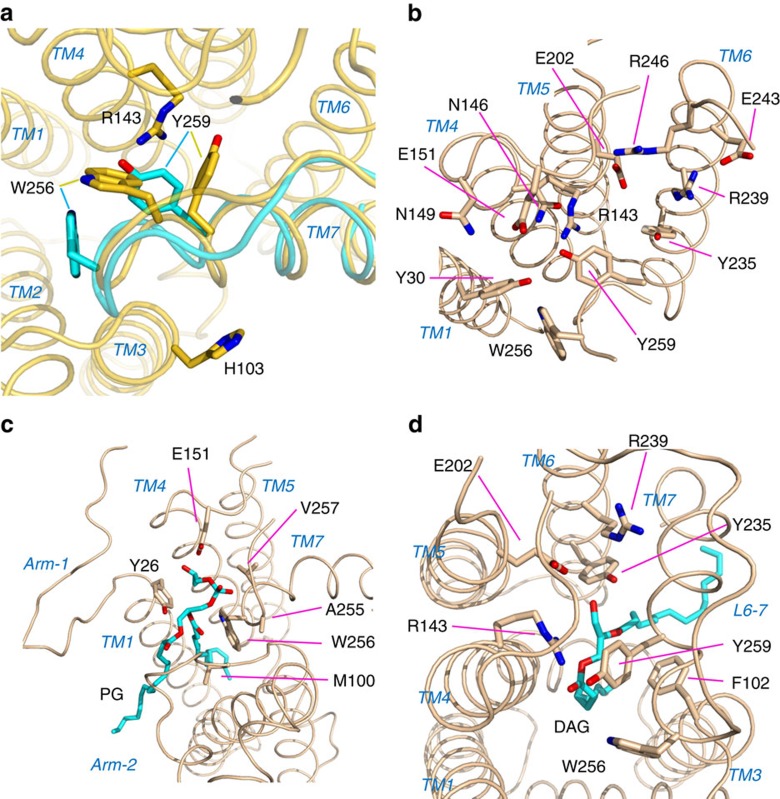
Structural features. (**a**) Conformational change around L6–7 between the two crystal forms. The form-1 structure (1.6 Å resolution) is shown in yellow, and form-2 (1.9 Å) in cyan. Only the loop L6–7 region shows significant conformational change, including sidechain rearrangement. This conformational change is hypothesized to be part of the transfer mechanism of the substrate PG from the first binding site to the second one. (**b**) Buried H-bond network. (**c**) PG-1 binding site. Backbone of PG is coloured in cyan. (**d**) DAG-2 binding site. The backbone of DAG is coloured in cyan.

The overall structure of Lgt represents a novel folding of 7-transmembrane (TM) proteins. Consistent with previous biochemical data as well as bioinformatic predictions^10^, the crystal structure of *Ec*Lgt contains seven TM helices (TMs 1–7; Fig. 2), which form the ‘body' (or TM core) of the protein structure. In addition, there are six β-strands (β1–β6) and four short helices (α1, η2, α3, α4). Between TMs 4 and 5, there is a ca. 50-residue insertion (that is, E151–P197), appearing as the ‘head' and being located in the periplasmic region. It possesses a globular shape and contains a twisted 4-strand β-sheet (β1, β5, β6 and β4) and two α-helices (α3 and α4; Fig. 2), with β1 contributed by the N terminus of the enzyme. Located N-terminally to the first transmembrane helix, TM1, is a β-hairpin arm formed by β2 and β3, termed ‘arm-1'. Between TMs 2 and 3, ∼20 residues that consist of α1 and η2 form a protruding structural moiety, referred to as ‘arm-2'. Both of these arms are amphipathic, with their hydrophobic sides facing the lipid bilayer. TMs 2 and 3, together with arm-2, form a small domain (referred to as the ‘minor TM domain') relatively independent from the rest of the TM core (referred to as the ‘major TM domain'). A central cavity exists between the minor and major TM domains. The N-terminal end of TM3 containing functionally important H^103^GGL^106^ motif^6^ is about 1–2 turn shorter than its neighbouring TMs 2 and 7, and appears to be below the level of membrane-periplasm interface. Interestingly, TM4 has the largest tilting angle (ca. 45°) relative to the direction of the membrane normal, and TM7 is highly curved at the N-terminal on the periplasmic side. Both TMs 4 and 7 contain Pro-induced kinks commonly found in membrane proteins of TM helices. The N-terminal of TM7, which follows the loop L6–7, is located in the ‘left shoulder' of the TM core. Part of this region (residues A255–Y259) showed a conformational change between the form-1 and form-2 crystal structures (Fig. 3a) as mentioned above. Essentially, the first turn of TM7 is lost in form-2, and side chains of both W256 and Y259 are re-positioned, with their largest shifts being 7.3 and 6.4 Å, respectively.

**Figure 4:**
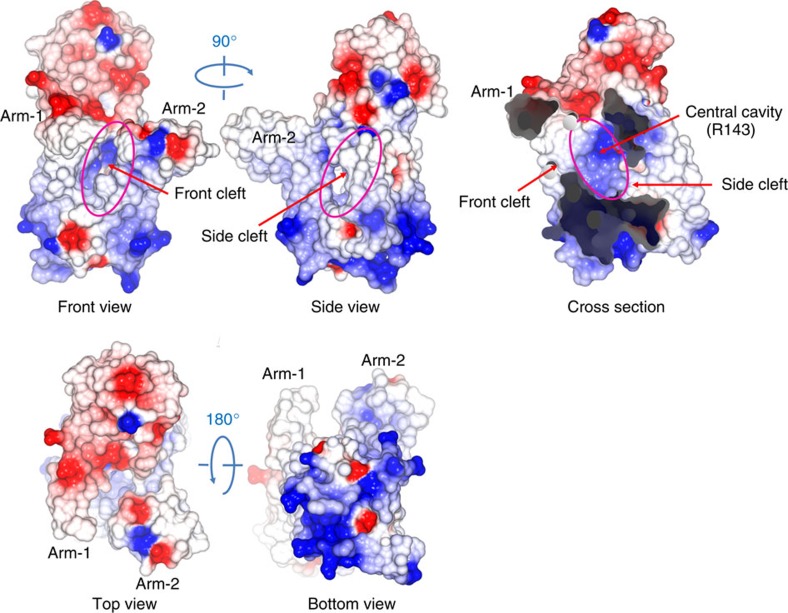
Surface presentation of electrostatic potential. Positively charged regions of the molecular surface of Lgt are shown in blue, and negatively charged regions in red. The distribution of charges satisfies the positive-inside rule of transmembrane protein topology. Structure presentations were generated using the program *Coot*.

The surface distribution of electrostatic potential of the Lgt molecule showed that the periplasmic side of the overall structure, including the head domain, is highly negatively charged (Fig. 4). The cytoplasmic side of the TM ‘body' domain is highly positively charged, consistent with the ‘positive-inside rule'^12^. The interior of the central cavity is mainly hydrophobic, with a patch of positively charged surface that is centred on R143^TM4^ and located below the membrane-periplasm interface level.

### Central cavity and the site of catalysis

Measured from the membrane surface level, the central cavity is about 20-Å deep, with a major opening to the periplasmic side (referred to as ‘periplasmic exit') and two clefts leading to the lipid region, one between the two arms (referred to as the ‘front cleft') and the other on the left side of the body (referred to as the ‘side cleft'; Fig. 2a). The bottom (that is, cytosolic side) of this cavity is completely hydrophobic, while the upper part (that is, periplasmic side) consists of more polar residues. All of these polar residues reside in the major TM domain, including the so-called signature motif of Lgt (G^142^RLGN FINGE LWG^154^)^10^. These polar residues participate in the formation of a conserved and partially buried hydrogen bond (H-bond) network formed by Y30^TM1^, R143^TM4^, N146^TM4^, N149^TM4^, E151, E202^TM5^, Y235^TM6^, R239^TM6^, E243^TM6^, R246^TM6^, W256^TM7^ and Y259^TM7^ (Fig. 3b). This network extends from the interior of the cavity to the periplasmic head domain. The hydrophobicity distribution inside the central cavity is consistent with the orientation of the donor-substrate PG, which embeds its two hydrophobic tails in the central part of the lipid bilayer, while protruding its more hydrophilic head group into the interface region between the outer leaflet of the inner membrane and periplasm. The positively charged patch at R143 embedded in a hydrophobic background (Fig. 4) may facilitate specific binding of the enzyme to the negatively charged donor-substrate PG. On the basis of these results, we propose that R143 is a critical residue of the catalytic centre of Lgt. Thus, the active site of the enzyme presumably faces the periplasm, with the PG, whose head group is specific for Lgt, approaching the vicinity of R143.

As mentioned above, there are two clefts in the central cavity connected to the membrane bilayer. The front cleft is about 6 Å wide, and is located between the two ‘arms' (in particular between TMs 1 and 2). The side cleft between the short TM3 from the minor TM domain and the highly curved TM7 from the major TM domain appears as a hole with a diameter of ca. 10 Å leading to the middle of the lipid bilayer. On the basis of these structural observations, we predict that the two clefts in Lgt are entrances for both substrates: PG and lipobox-containing peptide.

### Donor-substrate binding and specificity features

**Figure 5:**
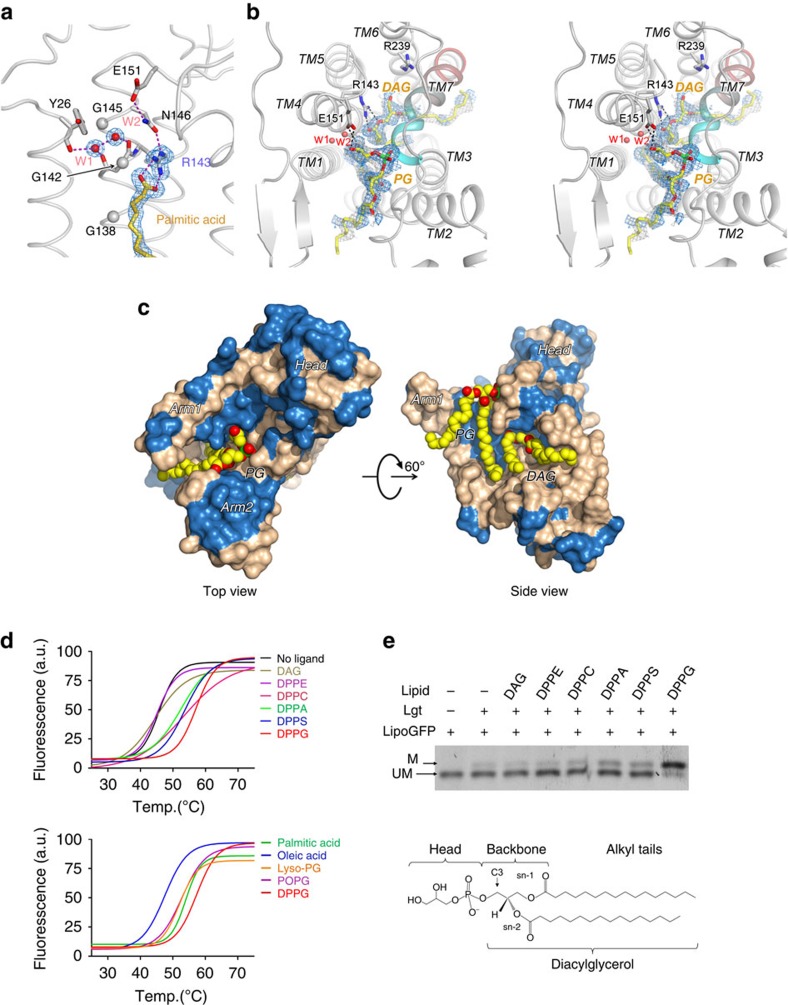
Binding site of donor-substrate PG. (**a**) Binding of palmitic acid to R143 in the form-1 crystal structure. The 2*F*_o_*−F*_c_ map contoured at 1.0 σ is shown around R143, palmitic acid, and two nearby water molecules (W1 and W2). (**b**) Stereo view of binding of PG and DAG to the first and second PG-binding sites in the form-2 crystal structure. The *F*_o_*−F*_c_ omit map (2.0 σ, blue) and 2*F*_o_*−F*_c_ omit map (1.0 σ, wheat colour) are shown around the ligands. TM7 is coloured from cyan at the N-terminal end to red at the C-terminal end. (**c**) PG binding. Lgt is shown in a molecular surface model, and PG and DAG are shown as a sphere model. Hydrophobic residues of Lgt are shown in wheat colour, and hydrophilic residues in blue. In the side view, the minor TM domain is removed for clarity. (**d**) Thermostability of Lgt in the presence of different lipids. Thermofluor data of (10 μM) WT Lgt in the presence of 100 μM of various lipids are fitted to a Boltzmann model. Chemical structures of the tested lipids (upper group) are shown in Supplementary Fig. 2a. The corresponding *T*_m_ values (average±s.d., in °C) are: blank, 46.0±0.2; DAG, 46±2; DPPE, 46±1; DPPC, 52±1; DPPA, 53±1; DPPS, 54±0.5; DPPG, 57±0.5; palmitic acid, 53±1; oleic acid, 48±0.5; lyso-PG, 52±0.5; and POPG, 53±0.5. All assays were repeated twice. (**e**) Lipid-donor specificity of WT Lgt. Conditions of the *in vitro* activity reactions were as following: 10 μM (final) Lgt, 200 μM lipoGFP, 1 mM specified lipids, at 37 °C for 90 min. The reaction mixtures were then subjected to SDS–PAGE followed by fluorescence imaging. Chemical structure of DPPG is illustrated, and that of others are shown in Supplementary Fig. 2a. Uncropped images are shown in Supplementary Fig. 6.

Comparison of the two crystal structures unveiled the binding modes of the donor substrate (PG), together with the molecular mechanisms underlying the diacylglyceryl transferase activity. In the form-1 *Ec*Lgt structure with a palmitic acid and an OG molecule, the guanidine group of R143 in the signature motif is found to bind with the carboxyl head-group of palmitic acid (Fig. 5a). The alkyl chain of the lipid molecule is located in the central cavity. The OG molecule is bound between the two arms. The two PG molecules found in this crystal structure are located outside the central cavity: one of them on the back side of the periplasmic head domain, and the other located outside of the side cleft, with its phosphoglycerol head group pointing to the cytoplasmic end of TM3. It is most likely that their presence contributed to proper crystal packing.

Analysis of the form-2 crystal structure showed that two lipid molecules are present in the central cavity (Fig. 5b,c), replacing OG and palmitic acid in the form-1 crystal. In the first PG-binding site (Fig. 3c), the side chain of E151 forms two H-bonds with the glycerol head group of PG. The negatively charged phosphate group of PG is bound to the N-terminal end of TM7 (that is, main chain amide groups of A255, W256 and V257) serving as its N-cap. It appears that the conformation of the L6–7 loop observed in form-2, which differs considerably from that of form-1, is crucial for the charge–helix dipole interaction. In this structure, the backbone glycerol of the bound PG molecule interacts with Y26 (important for the Lgt activity^10^) and arm-2. For example, the hydroxyl group of Y26 formed a H-bond with the ester oxygen of the sn-2 lipid chain, and the main chain amide group of M100 formed a H-bond with the sn-2 carbonyl oxygen. The second PG-binding site was observed near the catalytically essential residue R143 (Fig. 3d). Only the diacylglyceryl part of PG was observable. The glycerol moiety of PG resides in a pocket formed by F102, R143, E202, Y235, R239, W256 and Y259. This pocket was slightly larger than the corresponding pocket in the form-1 crystal, mainly due to the movement of the side chains of Y235, R239 and W256. For example, the side chain of R239 assumed dual conformations in the form-1 crystal, but only one conformation in the form-2 crystal. The side chain of W256 is packed with the periplasmic head domain in form-1, but protrudes into the cavity in form-2, where W256 separates the two PG-binding sites (Fig. 3a). Intriguingly, the leaving group of the substrate PG, glycerol-1-phosphate (G1P), was not observed. As it would not fit into the space of the binding pocket revealed in form-2, we hypothesize that the donor-substrate PG was hydrolysed to diacylglycerol (DAG) during the crystallization procedure. Accordingly, a DAG molecule was modelled into the second PG-binding site (Fig. 5b). The backbone glycerol of the DAG forms several H-bonds with Lgt, suggesting that binding of the DAG moiety is favourable for PG recognition. For example, the two oxygen atoms of sn-1 chain formed H-bonds with R143, similar to the interactions observed for the palmitic acid molecule bound in the form-1 crystal. The side chain hydroxyl group of Y235 formed a H-bond with the hydroxyl group of C3. R239 and E202 might also form water-mediated H-bonds with this hydroxyl group. Both alkyl chains of the second substrate ‘PG' molecule passed through the side cleft. We predict that the diacylglyceryl part of a native PG substrate uses a similar mode to bind into this site, while the loop L6–7 and N-terminal of TM7 seem to need a movement towards periplasm to make more room for the head group of the substrate PG.

To verify the significance of the lipid molecules observed in the crystal structures, a thermofluor assay^13^ was performed. Regarding the effects of diacylglyceryl phospholipids containing different head groups on the thermostability of Lgt, dipalmitoyl PG (DPPG) was found to increase the melting temperature (*T*_m_) by ∼10 °C, and was the most effective in stabilizing Lgt (Fig. 5d). In contrast, DAG, which lacks a head group, did not show any significant stabilizing effects. Other phospholipids tested showed stabilization effects between the two extreme cases of DPPG and DAG. Regarding the effects of saturation and number of the fatty acyl chains on the thermostability of Lgt, palmitic acid and PG with either two palmitoyl chains (DPPG) or single palmitoyl chain (lyso-PG), but not oleic acid (of an unsaturated (18:1 *cis*-9) alkyl chain), increased the *T*_m_ of *Ec*Lgt (Fig. 5d). In agreement with this observation, DPPG was found to be more effective in raising *T*_m_ than POPG (that is, 1-hexadecanoyl-2-(9Z-octadecenoyl)- sn-glycero-3-phospho- (1′-rac-glycerol) (16:0/18:1(9Z)), PG with one palmitoyl and one oleoyl chain). Together, phospholipids with saturated alkyl chains appeared to be more effective in stabilizing Lgt. Furthermore, an R143A point mutation variant displayed a similar phospholipid-binding pattern in the thermofluor assay (Supplementary Fig. 2b). Interestingly, the overall thermostability of R143A appears to be higher than that of WT Lgt, as measured by Δ*T*_m_∼8 °C. This observation implies that R143 is conserved for functional importance, rather than for structural reasons. This notion is further supported by the structural observation that the sidechain of R143^TM4^ is of a rare rotamer conformation, which is reinforced by a bulge near E206 in the middle of the neighbouring TM5. Together, it appears that G1P head group and the type as well as the number of fatty acyl chains contribute to the donor-substrate binding and the thermostability of Lgt.

### *In vitro* assay and substrate specificity

To correlate our structural observations with Lgt function, we analysed the enzymatic activity of recombinant *Ec*Lgt for different donor lipid substrates using a newly established *in vitro* lipoGFP assay (see Methods for details). As shown in Fig. 5e, DPPG was the most effective diacylglyceryl donor; dipalmitoyl phosphatidic acid (DPPA) and dipalmitoyl phosphatidyl-serine (DPPS), both negatively charged phospholipids also served as less-effective donors; neutral phospholipid, dipalmitoyl phosphatidyl-ethanolamine (DPPE), dipalmitoyl phosphatidyl-choline (DPPC) and DAG were not Lgt substrates. These observations are in agreement with the known specificity of Lgt for lipid substrate. In particular, DAG does not function as an intermediate lipid donor in the transacylation reaction. Regarding to fatty acyl chains, POPG is as effective in being a lipid donor when compared with DPPG (Supplementary Fig. 2c), which is in agreement with previous reports showing that fatty acyl composition of lipoproteins matches that of the phospholipids in the cell membrane and that both saturated and unsaturated fatty acyl chains are found in natural and recombinant lipoproteins^3^,^14^,^15^. Interestingly, lyso-PG was found to be a substrate of Lgt, although less effective than PG in transacylation (Supplementary Fig. 2c). In addition, our analysis confirmed that palmitic acid is an inhibitor of Lgt, and revealed that neither DAG nor oleic acid inhibit Lgt activity (Supplementary Fig. 2d). Hence, we propose that the crystal structure of form-1 represents a genuine Lgt-inhibitor complex.

The results of our *in vitro* assay also indicated that without additional substrate PG, the purified recombinant *Ec*Lgt was able to catalyse the transacylation reaction to the acceptor-substrate lipoGFP, provided that the molar concentration of Lgt is comparable to that of the polypeptide substrate (Fig. 1c). This observation suggests that PG is natively present in Lgt, as revealed by the crystal structure, and that prior binding of the acceptor prolipoprotein substrate is not required for the binding of the donor-substrate PG.

### *In vivo* complementation analysis of Lgt mutants

To correlate the structure with *in vivo* function of the Lgt enzyme, we complemented the *lgt*-conditional depletion strain of *E. coli* BW25113, referred to here as Δ*lgt*, separately with a number of Lgt variants. The point mutations were at residues known to be critical from earlier reports as well as deduced to be potentially important from the crystal structure. The mutant strain carried a pBAD/kana^R^-based rescuing plasmid that encodes an arabinose-inducible, myc and His6 double tagged WT Lgt, and grew normally on the inductive plate containing arabinose. However, it failed to form colonies (Fig. 1b, ‘vector') on repressive plate containing glucose, unless rescued by functional Lgt. Immunoblot analysis showed that the myc-tagged Lgt expressed well on the inductive plate, while no myc-tagged Lgt expression was detected on the repressive plate (Supplementary Fig. 3a). It was also shown that recombinant His-tagged WT *Ec*Lgt encoded by a pET28a-based plasmid was able to express in the Δ*lgt* cells, rescuing the Δ*lgt* strain either in the presence or absence of arabinose (Fig. 1b, ‘*Ec*Lgt'). Thus, the complementation system employed in this study was deemed suitable for studying the *in vivo* activity of Lgt variants.

Based on the new structural information as well as analysis of conserved residues, over 90 variants, including point mutations at over 40 distinct positions, deletions and insertions were constructed (Supplementary Fig. 3). These variants were then used in the complementation assay to map distribution of functionally important residues in the 3D (three-dimensional) structure (see Methods section for details).

**Figure 6:**
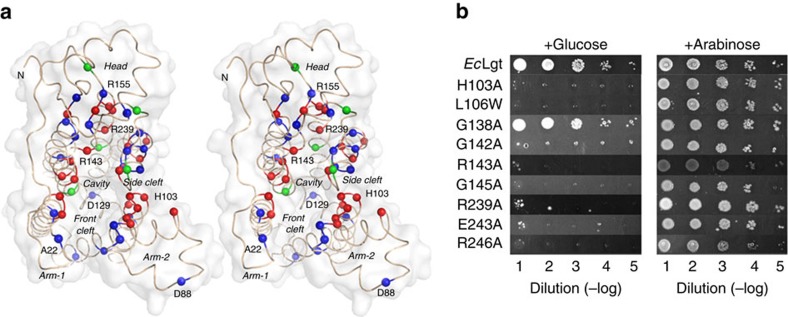
Mutational screening of Lgt structure. (**a**) Stereo top view of the Lgt backbone structure. Positions in which all mutations result in severe or total loss of activity are represented by red spheres; those in which all mutations showed little or no effect are represented by blue spheres; and others in which only some mutations lost activity are represented by green spheres. (**b**) Selected results of the complementation assay for Lgt variants (see Supplementary Fig. 3c for the complete set). For each variant of Lgt, a serial dilution of cell culture was spotted onto solid medium containing either 11 mM glucose (left columns) or 1 mM arabinose (right columns). Photos were taken 16 h after incubation at 37 °C. Appearance of colonies indicated functional Lgt.

This screening method provided us with a suitable tool to verify and analyse the structure–function aspects of our 3D structures (Fig. 6 and Supplementary Figs 3 and 4). The overall results of this screen are consistent with our structural observations, suggesting that the central cavity (including both the front and side clefts), the minor TM domain, and the buried H-bond network play essential roles in the transacylation activity of Lgt. In addition, we showed that inactive Lgt mutants (for example, R143A) can be overexpressed in the *E. coli* C43 strain. This observation confirms findings in a previous report, namely that there was no dominant-negative effect for Lgt^10^, and further suggests that the *in vivo* activity of Lgt does not require complex formation with other proteins.

## Discussion

Since its discovery in 1994, slow but steady progresses in the biochemical aspects of phosphatidylglycerol:prolipoprotein diacylglyceryl transferase (Lgt) have revealed interesting facets of this unique membrane transferase^16^,^17^,^18^. Here, we report a high-resolution crystal structure of *E. coli* Lgt, allowing for a structure–function correlation study of this ubiquitous diacylglyceryl transferase. The overall structure shows a unique folding, predicted to be conserved and classified into a characteristic superfamily. Its novel overall folding includes seven TM helices that form the minor and major TM domains, a periplasmic head domain, and two amphipathic arms associated with the two TM domains (Fig. 2). The central cavity located between the two TM domains has the essential features of the active site, with the two (front and side) clefts between the two TM domains serving as potential entrances for the substrates and the periplasmic exit for releasing the leaving group G1P. These structural features utilized for transferring diacylglyceryl from PG differ from other currently available structures of acyltransferases, for example, protein farnesyltransferase (FTase)^19^,^20^. While FTase is a water soluble, heterodimeric, zinc-dependent metalloenzyme, the membrane-located monomeric Lgt functions in presence of EDTA, suggesting differing catalytic reaction mechanisms.

Of the two crystal structures of Lgt, the form-1 is a complex of Lgt with the inhibitor palmitic acid; and the form-2 is a complex with the substrate PG and DAG (that is, the lipid moiety to be transferred). This study also permitted structure-based mutagenesis for identifying functionally important amino-acid residues. Results of the screening highlight three regions that are likely to play important roles in the transacylation reaction, namely the signature motif, a putative catalytic centre formed by a group of highly conserved residues (for example, R143 and R239) and the arm-2 region around the HGGL motif. On the basis of sequence analysis (Supplementary Fig. 1), we predict that the observed overall structure of *Ec*Lgt is conserved in other homologous Lgt proteins.

Availability of deduced Lgt sequences from a number of bacteria and limited mutation studies have allowed identification of some critical residues involved in the enzyme activity^10^. Our crystal structure provides direct explanations for these observations. The Lgt signature motif^10^ is located at the C-terminal three turns of the tilted TM4 helix and the following loop region, which is part of the periplasmic head domain. All the conserved polar residues in this motif are part of an extended H-bond network. In particular, absolutely conserved R143 directly binds the substrate PG in the second PG-binding site, and E151 binds directly to the substrate in the first PG-binding site. Therefore, we propose that the signature motif found in Lgt proteins is responsible for PG recognition, and the first PG-binding site functions as a substrate-selection filter.

**Figure 7:**
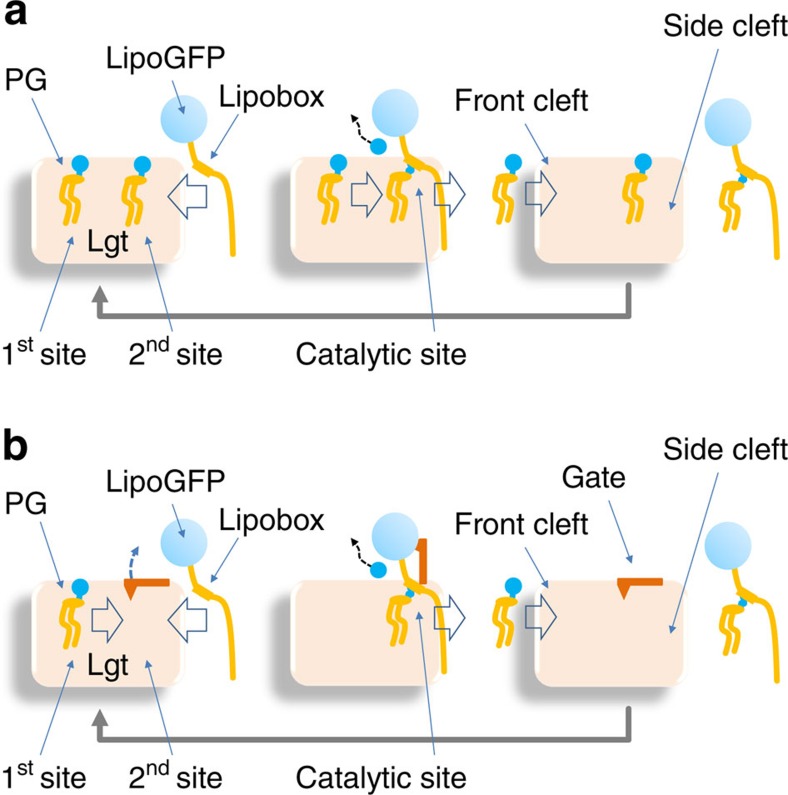
Two putative transacylation mechanisms of Lgt. In scheme (**a**), PG in its binding site II of the central cavity will come in contact with the lipobox of the peptide substrate bound to the H^103^GGL region in the central cavity for transfer of the diacylglyceryl moiety. G1P will leave through the periplasmic exit, and modified peptide substrate (product) will exit out of the side cleft. The PG molecule in binding site I will move to site II for the next round of catalysis, as just before the next prolipoprotein substrate moves in. In scheme (**b**), the conformational change seen in the loop connecting TM6 and TM7 helices on the periplasmic side suggests the presence of a gate (seen in form-2 crystal; shown in red) between PG-binding sites I and II. In this case, the conformational change, like induced-fit, will open the gate to allow PG in the binding site I to move to the site II and to take part in catalysis. After the product leaves, the gate will close again, and the cycle is repeated.

Based on the form-2 crystal structure, we further propose that the substrate PG slides from the first binding site into the second one (Fig. 7). Such a movement of the substrate PG seems to require a conformational change in the L6–7 loop region, which is likely to be even larger than what is observed when comparing the form-1 and -2 crystal structures. In the second binding site, the C3–O ester bond between the leaving group and C3 of diacylglyceryl moiety sits in a small pocket formed by the two highly conserved residues R143 and R239 as well as surrounding residues. The C3–O ester bond is activated in this pocket, ready to be cleaved on nucleophilic attack from the acceptor Cys of the lipobox-containing peptide substrate. The electron density that we observed in the second PG-binding site in the form-2 crystal was modelled as a DAG molecule, which was probably formed by slow hydrolysis during crystallization. Moreover, R239 is the most conserved residue in the Lgt homologues (Supplementary Fig. 1b). In the 3D structure, R239 locates close to R143, and is adjacent to two additional, highly conserved residues, namely E243 and R246 in the periplasmic head domain, where they form a H-bond network. In the form-2 structure, R239 forms a water-bridged H-bond with the hydroxyl group of the C3 of DAG. The fact that these residues are highly conserved across the entire Lgt family and are intolerant to mutations (Fig. 6) suggests that they play essential roles in the transacylation reaction. Together with R143, the R239 H-bond network is likely to catalyse the transfer of diacylglyceryl group to preprolipoprotein.

While the donor lipid substrate was clearly identified in our structures, the acceptor substrate, that is, the lipobox-containing polypeptide, was not part of the current structure. This is probably due to the lower *in vivo* binding affinities of the peptide substrates and of the products (that is, the lipid-modified polypeptides). Nevertheless, in *in vitro* assays, the peptide substrates appear to have ready access to Lgt, without the aid of additional proteins. In fact, our analysis provides strong evidence that by employing lipoGFP as a new model substrate, the diacylglyceryl-modified product can be experimentally pulled down with Lgt (Supplementary Fig. 5). This observation may be explained by the inability of the product to leave the enzyme in the absence of a pulling force provided by the hydrophobic membrane surroundings. Product inhibition reported previously *in vitro* kinetic study of Lgt^21^,^22^ would also support such a possibility. Future structure elucidation of such complexes will reveal further details of the function of Lgt.

For a typical signal peptide of a lipoprotein, its N-terminal region is probably directed towards the cytosol, with its hydrophobic region embedded in the membrane, and its C-terminal region with the lipobox oriented towards the periplasm. Our analysis indicates that the recombinant GST-lipoGFP protein serves as a substrate for Lgt, with or without removal of the N-terminal GST. This finding suggests that the N-terminal region of lipoGFP is not directly involved in the specific interaction with Lgt. This also accounts for the wide range of prolipoprotein signal sequences, from highly hydrophobic to distinctly hydrophilic, serving as a substrate for Lgt^23^. It has also been shown that, depending on the type (that is, fast or slow folding) of prolipoproteins, disruption of the Sec and/or Tat signal peptides results in defects in the transacylation process^17^,^18^,^24^,^25^. One possible interpretation is that to access the active site of Lgt, the lipobox-containing peptide has to be translocated to the periplasmic side of the membrane. Part of the binding site of the peptide substrate may be the N-terminal region of TM3, including the conserved HGGL motif^6^,^23^. H103 in the HGGL motif was identified as a critical residue for Lgt transacylation activity^6^, and we found that mutations in H103 as well as its surrounding residues result in loss of Lgt activity. Therefore, we propose that the lipobox-containing peptide substrates bind the HGGL motif from the side cleft, with the Cys residue pointing to the activated C3 carbon of diacylglyceryl moiety of PG bound in the second PG-binding site. Such peptide binding may facilitate the release of the proton, thus converting the thiol group into a thiyl radical. This thiyl radical then attacks the C3 atom of the C3–O ester bond, thus completing the transacylation reaction. The product diacylglyceryl prolipoprotein will then be released from the side cleft of the central cavity to the membrane bilayer, allowing the subsequent PG molecule to slide from the first binding site into the second site (Fig. 7a). A similar cooperative mechanism of substrate loading and product release was reported in the soluble acyltransferase FTase^19^.

As an alternative hypothesis for how Lgt catalyses the transacylation reaction, the two PG-binding sites may be occupied in an alternating manner: In one transacylation cycle, only one of the two PG-binding sites of Lgt may be occupied at any given time. The loop L6–7 and the N-terminal part of TM7 (including W256) function as a gate between the two sites, with the P269^TM7^-associated kink region functioning similarly to a gate hinge. On closure of the gate (as shown in the form-2 crystal structure) and completion of the first binding site, a PG molecule enters this first PG-binding site from the front cleft. On binding of the accepter-substrate peptide from the side cleft, the gate opens, and the donor-substrate PG slides from the first PG-binding site into the second one, which is the catalytic site. Considering its relatively small size in the closed state into which the G1P head group of PG would not fit, the catalytic site may only function on opening of the gate. Then, the peptide substrate approaches the PG molecule, followed by product release from the catalytic site via the same side cleft. The product release triggers the closing of the TM7 gate (Fig. 7b).

In conclusion, the crystal structures of Lgt reveal the molecular basis of several functional characteristics of this essential membrane enzyme. Novel findings include its overall transmembrane structure; PG-binding sites and the specificity-determining features; central cavity with clefts and opening for entry and exit of substrates and products; residues crucial to catalysis; possible transacylation mechanisms and inhibition by palmitic acid and product. This greater understanding of structural details, together with our findings from *in vitro* and *in vivo* functional studies, can now be exploited to develop potent inhibitors for next generation antibiotic leads and post-translational protein engineering. Our structural information has also created new possibilities for crystallizing Lgt proteins with their respective peptide substrates as well as products, facilitating better understanding of the molecular interactions with prolipoproteins.

## Methods

### Expression and purification

*Ec*Lgt gene was amplified using PCR from the genomic DNA of *E. coli* (BL21 strain)^26^. The amplified fragment was ligated into the *Nco*I and *Xho*I restriction sites of the expression vector pET-28a. The final construct, verified by sequencing, contained the Lgt-coding sequence fused to that of a C-terminal His-tag.

The recombinant plasmid was transformed into *E. coli* strain C43 (DE3) for expression. Cells were grown in Terrific Broth supplemented with 25 μg ml^−1^ kanamycin at 37 °C until OD_600 nm_ reached 0.6. Expression of recombinant *Ec*Lgt expression was induced by adding isopropyl β-D-1-thiogalactopyranoside (IPTG) to a final concentration of 0.5 mM. The cell culture was transferred to 16 °C and grown for 20 h. *E. coli* cells were collected by centrifugation at 4,000*g* for 30 min, resuspended in a lysis buffer (20 mM Tris-HCl (pH 8.0), 300 mM NaCl and 10% (v/v) glycerol), and then homogenized at 10,000–15,000 p.s.i. using aJN-R2C homogenizer (JNBio, China). After centrifugation at 18,000*g* (15 min and 4 °C), the supernatant was further ultracentrifuged at 100,000 *g* (1 h and 4 °C). The membrane fraction was collected and incubated at 4 °C for 1.5 h in 0.5% (w/v) *n*-decyl-β-D-maltopyranoside (DM; from Anatrace, USA). After another ultracentrifugation at 100,000*g* (40 min and 4 °C), the supernatant was purified using a HiTrap nickel column (GE Healthcare, USA). The His6-tagged protein sample was eluted with an elution buffer (20 mM Tris-HCl (pH 8.0), 300 mM NaCl, 10% (v/v) glycerol, 0.25% (w/v) DM and 300 mM imidazole) and further purified by size-exclusion chromatography (Superdex 200 from GE Healthcare) in a crystallization buffer (20 mM Tris-HCl (pH 7.5), 100 mM NaCl and 0.6% (w/v) OG (Anatrace)). The purified protein sample was concentrated to ca. 20 mg ml^−1^ using a 50-kDa cutoff Amicon Ultra-15 concentrator (Millipore, USA).

### *In vitro* activity assay

To study the activity of Lgt, we developed a GFP-based *in vitro* functional assay. First, pGEX-6p-1 based plasmid encoding a fusion protein, GST-PP-Lpp24-GFP (PP denotes the cleavage site recognized by PreScission Protease (PPase), and Lpp24 denotes the N-terminal 24 amino acid residues ‘MKATK LVLGAVILGSTLLAGC SSN' of Lpp, a major *in vivo* substrate of Lgt^5^, which includes the signal peptide with lipobox and three amino acids of the mature sequence), was constructed. Inclusion of the N-terminal GST effectively prevented transacylation during the expression of the fusion protein and PPase action resulted in prolipoGFP (referred to as lipoGFP) as the substrate for Lgt. A similar strategy of fusing Lpp24 peptide to *Shigella* apyrase as the reporter had been applied to study bacterial lipid modification of heterologous proteins *in vivo*^14^. In our case, *E.coli* C43 was transformed with the constructed plasmid and induced with 0.5 mM IPTG at 16 °C for 16 h. Cells were collected, resuspended in lysis buffer (20 mM Tris-HCl (pH 8.0), 300 mM NaCl, 10% (v/v) glycerol and 2 mM dithiothreitol (DTT), and then homogenized at 10,000–15,000 p.s.i. using a JN-R2C homogenizer. After centrifugation at 25,000*g* (30 min at 4 °C), the supernatant was purified using glutathione Sepharose 4B resin. PPase was then added to the column and incubated for 16 h at 4 °C. The resulting Lpp24-GFP fusion protein (MW: ∼29 kDa), referred to as lipoGFP, was eluted with the lysis buffer, with PPase remaining bound in the column. The lipoGFP sample was further purified by size-exclusion chromatography (Superdex 200, GE Healthcare) in a storage buffer (20 mM Tris-HCl (pH 8.0), 100 mM NaCl and 2 mM DTT). The purified protein substrate was concentrated to ca. 15 mg ml^−1^, and then stored at −80 °C until further use.

In a typical *in vitro* transacylation assay, *Ec*Lgt (enzyme, 1.2 μM) was incubated with lipoGFP (acceptor substrate, 7 μM) in reaction buffer (20 mM Tris (pH 8.0), 4 mM DTT and 5 mM EDTA) in the presence (40 μM) or absence of PG (donor substrate) at 37 °C for 5–20 min. Reaction mixture containing 5 ng lipoGFP was loaded to SDS–PAGE gel (10%) and subjected to electrophoresis. The gel was scanned with Typhoon FLA 7000 (GE Healthcare), and fluorescent signals were recorded for both lipid-modified and unmodified lipoGFP. All lipids that were added to these assays were purchased from Avanti Polar Lipids Inc.

### Crystallization

Before crystallization, the concentrated pure Lgt preparation in crystallization buffer was mixed with PG (Avanti, USA) and palmitic acid (Avanti) at molar ratio 1:6:3 so that the final protein concentration was ca. 10–15 mg ml^−1^. Crystallization was carried out at 16 °C using the hanging drop vapour diffusion method. Crystals of Lgt were obtained with a precipitation solution of 0.1 M Tris (pH 6.5), 0.1 M CaCl_2_ and 13% (w/v) PEG2000 MME. Crystals appeared in 1 week, and grew to a full size of 0.1 × 0.1 × 0.05 mm in 15–20 days. Crystals were flash-cooled and stored in liquid nitrogen. Seleno-L-methionine derivative crystals were grown under conditions identical to those used for the native ones.

### Structure determination

Diffraction data were collected at the Shanghai Synchrotron Radiation Facility (SSFR) beamline BL17U and Photon Factory (Japan) beamline BL1A, and were then processed with the program HKL2000 (ref. 27). Further processing was carried out using programs from the CCP4 suite^28^. The diffraction resolutions of the native and seleno-L-methionine derivative Lgt were up to 1.6 and 2.0 Å, respectively.

The selenium sites were identified with Shelx-D^29^ using the single wavelength anomalous dispersion method. The sites were refined, and phases were calculated with the program Phenix^30^. The initial model was built using the program Phenix.autobuild. The model was improved by iterative cycles of manual fitting using COOT^31^ and refinement using Phenix.refine. Ligand, detergent and water molecules were added to the model at later stages of the refinement based on clear non-protein densities. Model validation was carried out using the Molprobity server^32^.

### Plate complementation assay

A null allele of Lgt in the strain BW25113 carrying inducible WT *Ec*Lgt gene on a modified pBAD plasmid was constructed. This BW25113/Δ*lgt*-pBAD/kana^R^-Lgt strain is referred to as Δ*lgt*. Since *lgt* is an essential gene for the survival of *E. coli*, the supplemented WT *Ec*Lgt gene carried in the pBAD plasmid was necessary for the construction of Δ*lgt*. This gene was kept in the silent mode by using glucose when Lgt variants were tested. The knockout of Lgt from the genome was carried out following a previous report^10^. The pBAD-Lgt plasmid was modified from amp^R^ to kana^R^ to make it compatible with the Datsenko–Wanner knockout system^33^. This plasmid expressed functional WT Lgt on induction with arabinose, and was used to rescue the lethal Lgt knockout whenever necessary. Addition of 0.2% (11 mM) glucose in LB medium strictly restrained the expression of Lgt from the pBAD-Lgt plasmid. To be compatible with the kana^R^ pBAD-Lgt plasmid, the pET28a vector was also modified from kana^R^ to amp^R^, and its replication origin, pBR322, was replaced with p15a. This vector was used to carry Lgt variant genes in complementation assays. The Lgt variants constructed in pET28a/amp^R^ showed leaky expression in cells of the ΔLgt strain. Since the BW25113 strain is not compatible with IPTG induction, what rescued Δ*lgt* was the leaky WT expression in the absence of IPTG (Supplementary Fig. 3a), as was shown in a previous report^10^. In addition, the Lgt protein encoded by the pBAD-Lgt plasmid was fused with a myc-His6 double tag at its C terminus, while that encoded by the pET28a plasmid was fused only with C-terminal His6-tag.

The plate complementation assay was performed as follows. The plasmid pET28a encoding an Lgt variant was transformed into the Δ*lgt* strain. The cells were then grown at 37 °C in (5 ml) LB medium supplemented with 100 μg ml^−1^ ampicillin, 25 μg ml^−1^ kanamycin and 1 mM arabinose. When OD_600_ reached 0.6, the cell culture was inoculated in fresh LB medium without arabinose in a volume ratio of 1:100 for 2 h at 37 °C (OD_600_ of ∼0.8–1.0), to reduce the effect of arabinose on the subsequent plate complementation assay. Cells were centrifuged and resuspended in LB to adjust their OD_600_ to1.0. Suspensions of all constructs were sequentially diluted 10–100,000 folds. A measure of 0.5 μl of each dilution was spotted on the solid medium in plates supplemented with 100 μg ml^−1^ ampicillin, 25 μg ml^−1^ kanamycin and either 1 mM arabinose or 11 mM glucose. After 16 h incubation at 37 °C, colony formation was recorded as an indication of transacylation activity of an Lgt variant. The complementation assays of all the variants were repeated at least three times, and the results showed good reproducibility. Since knockout of Lgt is lethal, the leaky expression levels (that is, in the absence of IPTG) of Lgt variants could not be verified in the Δ*lgt* strain; instead, they were verified in the parental BW25113 strain. In this case, membrane fractions of cell lysates were collected and subjected to SDS–PAGE and anti-His immunoblotting. Expression levels of selected inactive mutations were verified using immunoblotting. Our results for all tested cases showed that the expression levels were comparable to that of WT (Supplementary Fig. 3b).

Arm-1 connects the N-terminal β1 strand from the head domain with the TM1 helix of the TM core. For analysis of arm-1, an N-terminal truncation mutant (ΔN-9) and a deletion variant of arm-1 (Δarm1) were constructed. Of these, Δarm1, in which residues S17, I18, V21 and A22 were deleted, while still containing G19 and P20 at the β-hairpin tip, had no significant effect on the activity of Lgt, that is, colonies were formed normally in the complementation assay (Supplementary Figs 3c and 4a). In contrast, the truncation variant, ΔN-9, in which nine residues were removed from the N terminus (including the β1 strand), showed no activity. These results suggested that β1 and proper connection between β1 and the arm-1 is critical for the activity of Lgt, not however, the length of arm-1. To test the hypothesis whether arm-1 forms an extended β-sheet with the peptide substrate, we constructed the mutant A22P. Such a variant would abolish two H-bonds between the arm-1 and the putative peptide substrate. However, complementation assay showed no deleterious effect of A22P on Lgt activity. Similarly, H24A mutation also did not affect the activity. Hence it is concluded that arm-1 is unlikely to be directly involved in the interaction with the acceptor-substrate peptide via β-sheet extension.

For analysis of arm-2, we constructed the mutants G98P, G99P and M100W, all of which face the central cavity. Specifically, M100W was designed to block the entrance of the front cleft, and the other two mutations were designed to disrupt potential binding with substrate PG or peptide. Results of the complementation assay showed that while G98P mutation showed no effect on Lgt activity, G99P and M100W mutants lost activity completely (Supplementary Figs 3c and 4b). These results are in agreement with the structural observation that G99 and M100 are involved in the first PG-binding site in the form-2 crystal. D88N (which breaks a salt-bridge bond with R94) and D97N (which breaks another salt bridge with R73) mutations had no effect on Lgt activity, suggesting that although these salt bridges might contribute to the structural stability of the minor TM domain, individually they are of no functional significance. Taken together, arm-2 (in particular G99 and M100) appears to be involved in the binding of the donor substrate to the first PG-binding site in the front cleft.

In our analysis of residues located within the central cavity, point mutants R143A/E/K were constructed. All these mutants lost activity (Fig. 6, Supplementary Figs 3c and 4c). Also, inside the central cavity, mutations Y26A/F/Q and G27L/Q/W in TM1, G138I/V (but not G138A), G142A/I/V, G145A/I/V and E151A/Q in the signature motif all resulted in inactive enzymes. This result is consistent with the notion that the central cavity is essential for substrate binding and catalysis.

In the side cleft of the central cavity (that is, the region between TMs 3 and 7), the mutation L106W in the upper (periplasmic) half of TM3 and M262Q/Y in TM7 resulted in inactive Lgt (Fig. 6, Supplementary Figs 3c and 4b). In contrast, I110W, whose position is located just one helical turn away from L106 towards the cytosolic side, maintained the activity. These results suggest a possibility that the upper part of this cleft may serve as a substrate entrance. Near this region, G263A/L/V at a position facing the buried H-bond network (involving R239 in particular) and P269A (disrupting the Pro-induced kink in TM7) also lost activity. The structural flexibility of TM7 caused by the P269-induced kink is probably essential for the conformational change observed in the region of loop L6–7, and for the PG transfer from the first binding site to the second one. In contrast, Q264A at a solvent exposed position showed no effect on the activity. Taken together, these observations suggest that the side cleft, including the local conformation in TM7, is important for the activity.

To confirm the importance of H103 at the N-terminal end of TM3, previously identified as being essential for Lgt activity^6^, H103A/N/Q/R/Y mutations were introduced. None of the mutants could complement, indicating the essential role of this residue in Lgt activity (Fig. 6, Supplementary Figs 3c and 4b). To further test the importance of the micro-environment of H103, several residues that interact with H103, were also mutated. As shown in our results, the mutants Y80F/Q, S101D and F102A/W, all mutations in the minor TM domain, lost their activity, while TM-7 mutant variants Q258A and S261A maintained their activity (Supplementary Fig. 3c). Together, these observations suggest that interactions of H103 with residues of the minor TM domain are important for activity, while interactions with residues from the major TM domain are not. This notion is in agreement with the structural observation that the form-1 and form-2 crystals exhibit two different conformations at the interface between TM3 and TM7.

To investigate the functional roles of the remaining periplasmic head domain, the mutant variantsY235F/L/S/T, R239A/E/H/K/Q, E243A/Q/R and R246A/E/H/K/Q (all located in the buried H-bond network) were constructed. Our results showed that none of these mutants could complement, except for Y235T, which displayed partial activity (Fig. 6, Supplementary Figs 3c and 4c). In contrast, other two mutants in the network, namely E202Q and Y259A/E/F, maintained good activity, while E202A/L exhibited partial activity. Mutation G154A (which is buried in the core of the head domain) and P197A both let to severe loss of activity, most likely for structural reasons. In contrast, E172Q (partially solvent exposed) maintained the activity. To probe the structurally observed PG-binding site on the back of the head domain, we constructed mutant variants R155A/Q, D157A/N, H196A/Q and Y201F. All of these variants maintained colony-formation activity. Thus, this region is unlikely to be involved in the formation of the active site, and the observed binding of PG may in fact be a crystallization artefact.

Although the L6–7 region (that is, residues 247–256) is not conserved (Supplementary Fig. 1), its conformational change (Fig. 3) as well as its positional proximity to both H103 and R143 prompted us to perform a mutational analysis on this loop. Among the mutants constructed, P248A, F252L and T253L, but not T253N, maintained activity (Supplementary Fig. 3c). W256A as well as two insertion variants that contained either one extra Gly residue (labelled as IN-G in Supplementary Fig. 3c) or a Gly-Ser pair (IN-GS) between F252 and T253 lost activity only partially. Together, the middle part of L6–7 appears to be less sensitive to structural perturbation, as measured by its impact on activity.

On the cytosolic side of the TM core resides D129, one of the most conserved acidic residues which forms a charge–helix dipole interaction with the N-terminal of TM6. However, analysis of the mutant form D129A/N showed that it is non-essential for full activity (Supplementary Fig. 3c). Similarly, E56 of TM2 forms a salt-bridge bond with R122 of TM4; yet, mutation E56Q showed no effect on the activity. Therefore, the cytosolic side of the TM core seems to have no significant functional roles in Lgt catalysis.

### Thermostability assay

To evaluate the effects of mutations and lipid binding on stability of *Ec*Lgt, thermofluor analysis was performed using a quantitative PCR instrument (Rotor-Gene 6600, Corbett Research, Australia). As temperature rose, thiol-specific fluorochrome *N*-[4-7-(diethylamino-4-methyl-3-coumarinyl)-phenyl]-maleimide (CPM from Invitrogen, USA) was used as fluorescence probe to monitor the conformational change. CPM fluorescence signal was measured with a 387-nm excitation and a 463-nm emission. Since WT *Ec*Lgt does not have Cys residues, Cys was introduced into a buried position in the minor TM domain, G64^TM2^, and this G64C mutant was used as the background for constructing variants to be used in the thermofluor analysis. The purified protein of *Ec*Lgt-G64C or its variants (10 μg μl^−1^) were added into reaction buffer (100 mM MES (pH 6.0), 100 mM NaCl and 25 μM CPM) in the presence and absence of 0.1 mM lipids analysed. Reaction volume was 20 μl, and the final protein concentration was 0.4 μg μl^−1^. The quantitative PCR instrument was programed to increase temperature by 1 °C in 1 min and then to stay at each temperature for 1.5 min between 25 °C and 95 °C. Data analyses were performed using the program Graphpad Prism.

### Pull-down assay

To further analyse the effects of point mutations, we performed an affinity assay using His-tagged Lgt variants to pull down the model substrate, lipoGFP. Plasmids pET28a encoding Lgt variants with a C-terminal His6-tag were transformed into *E. coli* strain C43 (DE3). Then the cells (5 ml each) were grown at 37 °C in LB medium supplemented with 100 μg ml^−1^ ampicillin. When OD_600 nm_ reached 0.6, protein expression was induced with 0.5 mM IPTG. The cell culture was transferred to 16 °C and grown for 14 h. The cells were collected and resuspended in 1.5 ml lysis buffer and homogenized with a Sonic Dismembrator-550 (Fisher Scientific). After centrifugation at 13,000*g* (10 min and 4 °C) to remove cell debris, the supernatant was collected and incubated with 0.6% (w/v) OG for 1 h at 4 °C. The solubilized Lgt preparation was then purified using a Cobalt resin column (20 μl). The resin was washed with ∼100 μl wash buffer (20 mM Tris-HCl (pH 8.0), 300 mM NaCl, 10% (v/v) glycerol, 0.6% (w/v) OG and 15 mM imidazole). Last, the resin was incubated for 30 min with excess (∼100 μM final) of either lipoGFP or the lipoGFP(C21S) mutant. The sample was further washed three times with the wash buffer to remove non-specific binding of lipoGFP. The resin (4 μl) was loaded to SDS–PAGE gel (16%) and subjected to electrophoresis. The gel was imaged with ChamGel-5000 (Sage Creation, China) to record the fluorescence signal of GFP before staining with Coomassie brilliant blue.

## Additional information

**How to cite this article:** Mao, G. *et al.* Crystal structure of *E. coli* lipoprotein diacylglyceryl transferase. *Nat. Commun.* 7:10198 doi: 10.1038/ncomms10198 (2016).

## Supplementary Material

Supplementary Information Supplementary Figures 1-7 and Supplementary References.

## Figures and Tables

**Table 1:** Statistics of data collection and refinement.

	**Native (isoform 1)**	**Native (isoform 2)**	**Se (peak)**
*Data processing*			
Wavelength (Å)	0.9200	0.9791	0.9787
Space group	P2_1_2_1_2_1_	P2_1_2_1_2_1_	P2_1_2_1_2_1_
Cell dimensions *a, b, c* (Å)	50.4, 61.0, 117.8	50.1, 60.9, 117.4	50.5, 61.2, 118.0
Resolution (Å)	50–1.6 (1.7–1.6)*	40–1.9 (2.0–1.9)	50–1.8 (1.9–1.8)
Completeness (%)	99.9 (99.1)	98.3 (98.2)	96.5 (97.4)
*R*_merge_ (%)†	13.3 (>100)	10.1 (>100)	13.6 (99.2)
*I*/σ(*I*)	36.9 (1.9)	19.3 (3.9)	11.6 (2.0)
Unique reflections	49,244 (4,815)	28,535 (2,819)	33,589 (3,318)
Redundancy	12.3 (8.6)	9.0 (7.9)	6.9 (7.6)
			
*Refinement*			
Resolution (Å)	32.4–1.6	27.5–1.9	
No. of reflections (test)	49,147 (2,500)	28,162 (1,425)	
*R*_work_/*R*_free_ (%)‡	17.9/22.2	21.2/25.0	
*Number of atoms*			
Protein	2,371	2,365	
Lipid	237	223	
Water	103	130	
*Average B-factor (Å*^*2*^)			
Protein	19	34	
Lipid	47	52	
Water	29	43	
*r.m.s. deviations*			
Bond lengths (Å)	0.006	0.007	
Bond angles (°)	1.035	1.108	
*Ramachandran plot (%)*§			
Favoured region	98.7	95.6	
Allowed region	1.3	4.4	

^*^Values in parentheses are for the highest resolution shell.

^†^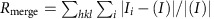 where *I*_*i*_ is the intensity for the *i-*th measurement of an equivalent reflection with indices *h*, *k* and *l*.

^‡^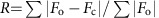 where *F*_o_ and *F*_c_ are the observed and calculated structure factors, respectively.

^§^Calculated using *MolProbity*.
