# Supplementary material for: Crystal structure of E. coli lipoprotein diacylglyceryl transferase
Source: Nat Commun. 2016 Jan 5;7:10198. doi: 10.1038/ncomms10198 (PMC4728403; doi:10.1038/ncomms10198)
Supplement: Supplementary Information — Supplementary Figures 1-7 and Supplementary References. [file ncomms10198-s1.pdf]

a

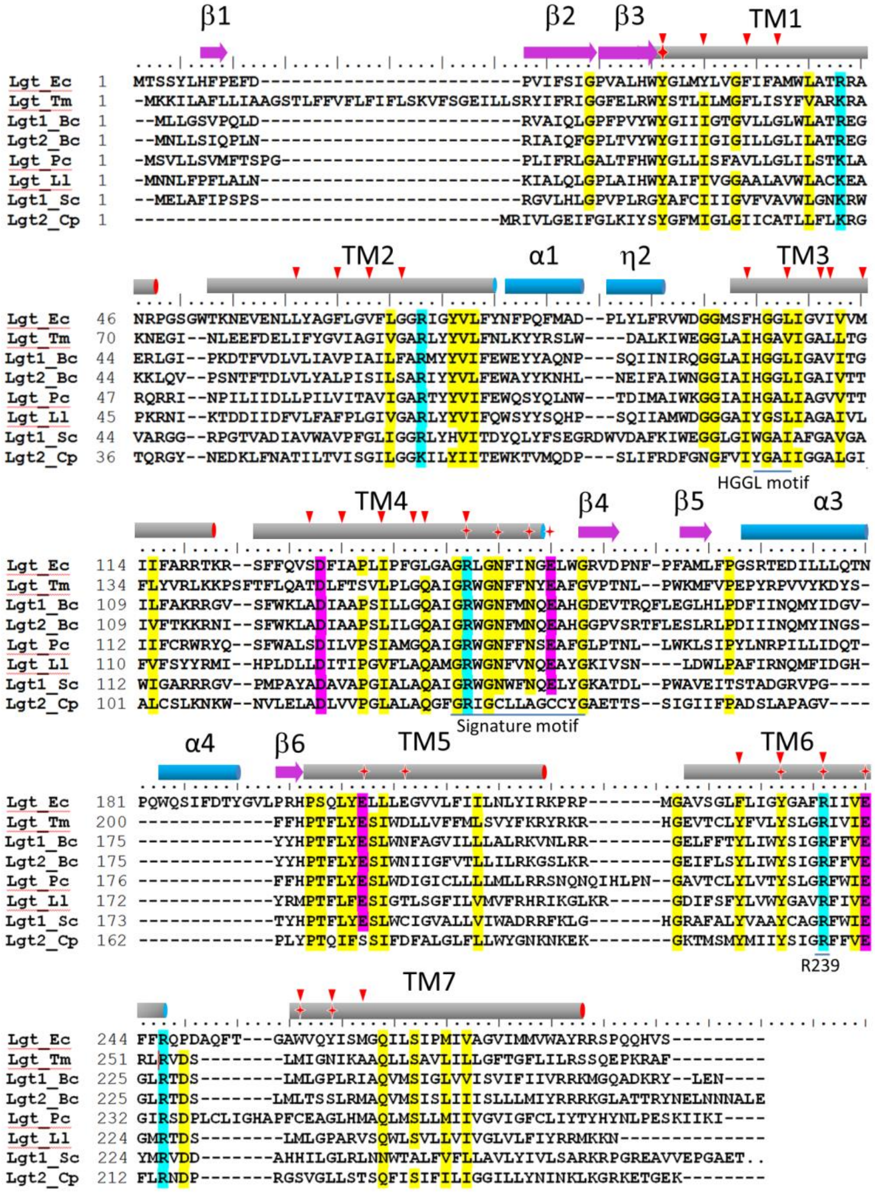
β1
β2
β3
TM1
Lgt_Ec 1 MTSSYLHFPEFD-------------------------PVIFSIGPVALHWYGLMYLVGFIFAMWLATRRA
Lgt_Tm 1 -MKKILAFLLIAAGSTLFFVFLFIFLSKVFSGEILLSRYIFRIGGFELRWYSTLIILMGFLISYFVARKRA
Lgt1_Bc 1 --MLLGSVPQLD-------------------------RVAIQLGPFPVYWYGIIIGTGVLLGLWLATREG
Lgt2_Bc 1 --MNLLSIQPLN-------------------------RIAIQFGPLTVYWYGIIIGIGILLGLILATREG
Lgt_Pc 1 -MSVLLSVMFTSPG-----------------------PLIFRLGALTFHWYGLLISFAVLLGLILSTKLA
Lgt_Ll 1 -MNNLFPFLALN-------------------------KIALQLGPLAIHWYAIFIVGGAALAVWLACKEA
Lgt1_Sc 1 --MELAFIPSPS-------------------------RGVLHLGPVPLRGYAFCIIIGVFVAVWLGNKRW
Lgt2_Cp 1 ---------------------------------MRIVLGEIFGLKIYSYGFMIGLGIICATLLFLKRG
TM2
α1
η2
TM3
Lgt_Ec 46 NRPGSGWTKNEVENLLYAGFLGVFLGGRIGYVLFYNFPQFMAD--PLYLFRVWDGGMSFHGGLIGVIVVM
Lgt_Tm 70 KNEGI--NLEEFDELIFYGVIAGIVGARLYYVLFNLKYYRSLW----DALKIWEGGLAIHGAVIGALLTG
Lgt1_Bc 44 ERLGI--PKDTFVDLVLIAVPIAILFARMYYVIFEWEYYAQNP---SQIINIRQGGLAIHGGLIGAVITG
Lgt2_Bc 44 KKLQV--PSNTFTDLVLYALPISILSARIYYVLFEWAYYKNHL---NEIFAIWNGGIAIHGGLIGAIVTT
Lgt_Pc 47 RQRRI--NPILIIDLLPILVITAVIGARTYYVIFEWQSYQLNW---TDIMAIWKGGIAIHGALIAGVVTT
Lgt_Ll 45 PKRNI--KTDDIIDFVLFAFPLGIVGARLYYVIFQWSYYSQHP---SQIIAMWDGGGAIYGSLIAGAIVL
Lgt1_Sc 44 VARGG--RPGTVADIAVWAVPFGLIGGRLYHVITDYQLYFSEGRDWVDAFKIWEGGLGIWGAIAFGAVGA
Lgt2_Cp 36 TQRGY--NEDKLFNATILTVISGILGGKILYIITEWKTVMQDP---SLIFRDFGNGFVIYGAIIGGALGI
HGGL motif
TM4
β4
β5
α3
Lgt_Ec 114 IIFARRTKR--SFFQVSDFIAPLIPFGLGAGRLGNFINGELWGRVDPNF-PFAMLFPGSRTEDILLLQTN
Lgt_Tm 134 FLYVRLKKPSFTFLQATDLFTSVLPLGQAIGRWGNFFNYEAFGVPTNL--PWKMFVPEPYRPVVYKDYS-
Lgt1_Bc 109 ILFAKRRGV--SFWKLADIAAPSILLGQAIGRWGNFMNQEAHGDEVTRQFLEGLHLPDFIINQMYIDGV-
Lgt2_Bc 109 IVFTKKRNI--SFWKLADIAAPSLILGQAIGRWGNFMNQEAHGGPVSRTFLESLRLPDIIINQMYINGS-
Lgt_Pc 112 IIFCRWRYQ--SFWALSDILVPSIAMGQAIGRWGNFFNSEAFGLPTNL--LWKLSIPYLNRPILLIDQT-
Lgt_Ll 110 FVFSYYRMI--HPLDLLDITIPGVFLAQAMGRWGNFVNQEAYGKIVSN----LDWLPAFIRNQMFIDGH-
Lgt1_Sc 112 WIGARRRGV--PMPAYADAVAPGIALAQAIGRWGNWFNQELYGKATDL--PWAVEITSTADGRVPG----
Lgt2_Cp 101 ALCSLKNKW--NVLELADLVVPGLALAQGFGRIGCLLAGCCYGAETTS--SIGIIFPADSLAPAGV----
Signature motif
α4
β6
TM5
TM6
Lgt_Ec 181 PQWQSIFDTYGVLPRHPSQLYELLLEGVVLFIILNLYIRKPRP-------MGAVSGLFLIGYGAFRIIVE
Lgt_Tm 200 -------------FFHPTFLYESIWDLLVFFMLSVYFKRYRKR-------HGEVTCLYFVLYSLGRIVIE
Lgt1_Bc 175 -------------YYHPTFLYESLWNFAGVILLLALRKVNLRR--------GELFFTYLIWYSIGRFFVE
Lgt2_Bc 175 -------------YYHPTFLYESIWNIIGFVTLLILRKGSLKR--------GEIFLSYLIWYSIGRFFVE
Lgt_Pc 176 -------------FFHPTFLYESLWDIGICLLLLMLLRRSNQNQIHLPN--GAVTCLYLVTYSLGRFWIE
Lgt_Ll 172 -------------YRMPTFLFESIGTLSGFILVMVFRHRIKGLKR------GDIFSFYLVWYGAVRFIVE
Lgt1_Sc 173 -------------TYHPTFLYESLWCIGVALLVIWADRRFKLG-------HGRAFALYVAAYCAGRFWIE
Lgt2_Cp 162 -------------PLYPTQIFSSIFDFALGLFLLWYGNKNKEK--------GKTMSMYMIIYSIGRFFVE
R239
TM7
Lgt_Ec 244 FFRQPDAQFT---GAWVQYISMGQILSIPMIVAGVIMMVWAYRRSPQQHVS---------
Lgt_Tm 251 RLRVDS--------LMIGNIKAAQLLSAVLILLGFTGFLILRSSQEPKRAF---------
Lgt1_Bc 225 GLRTDS--------LMLGPLRIAQVMSIGLVVISVIFIIVRRKMGQADKRY--LEN----
Lgt2_Bc 225 GLRTDS-------LMLTSSLRMAQVMSISLIIISLLLMIYRRRKGLATTRYNELNNNALE
Lgt_Pc 232 GIRSDPLCLIGHAPFCEAGLHMAQLMSLLMIIVGVIGFCLIYTYHYNLPESKIIKI----
Lgt_Ll 224 GMRTDS--------LMLGPARVSQWLSVLLVIVGLVLFIYRRMKKN--------------
Lgt1_Sc 224 YMRVDD-------AHHILGLRLNNWTALFVFLLAVLYIVLSARKRPGREAVVEPGAET..
Lgt2_Cp 212 FLRNDP-------RGSVGLLSTSQFISIFILIGGILLYNINKLKGRKETGEK--------

b

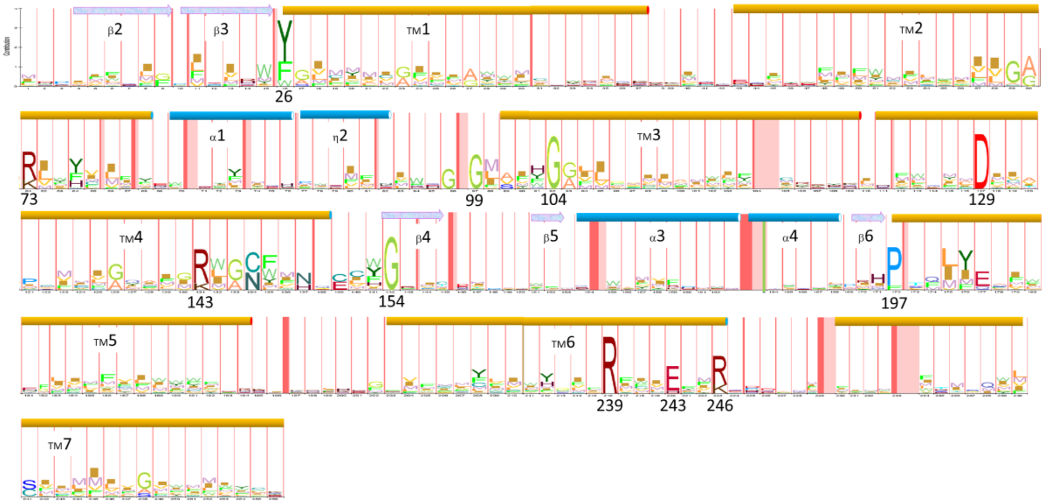

Supplementary Figure 1. Analysis on amino acid sequences of Lgt proteins.

a. Amino acid sequence alignment of Lgt proteins from *E. coli* (Lgt_Ec), *Thermotoga maritima* (Lgt_Tm), *Bacillus cereus* (Lgt1_Bc and Lgt2_Bc), *Paulinella chromatophora* (Lgt_Pc, a freshwater filose amoeba), *Lactococcus lactis* (Lgt_Ll), *Streptomyces coelicolor* (Lgt1_Sc), and *Clostridium perfringens* (Lgt2_Cp) are included. Secondary structural elements *of Ec*Lgt are marked on the top, including β1: L6–F8; β2: P13–G19; β3: P20–W25; TM1: H24–R47; TM2: T53–N81; α1: N81–D88; η2: V96–G99; TM3: S101–T120; TM4: S123–G150; β4: G154–D157; β5: A163–L165; α3: S169–N180; α4: W183–Y190; β6: P194–H196; TM5: P197–R219; TM6: A226–F245; and TM7: G254–R284. Residues that make up the interior surface of the central cavity are marked with red triangles, and residues that participate in the buried H-bond network are marked with red stars. Conservative positions are highlighted. Sequence alignment was performed using the program ClustalX and formatted with BioEdit. b. WebLogo representation of Lgt homologs. It was generated by the PFAM web-server [1] using *Ec*Lgt as the template (number in seed: 67; number in full: 5002). The larger the letter is at a given position, the more conservative the position is. Pink regions indicate potential insertions. The residue numbers at the bottom are from *Ec*Lgt. Secondary structures are marked on the top.

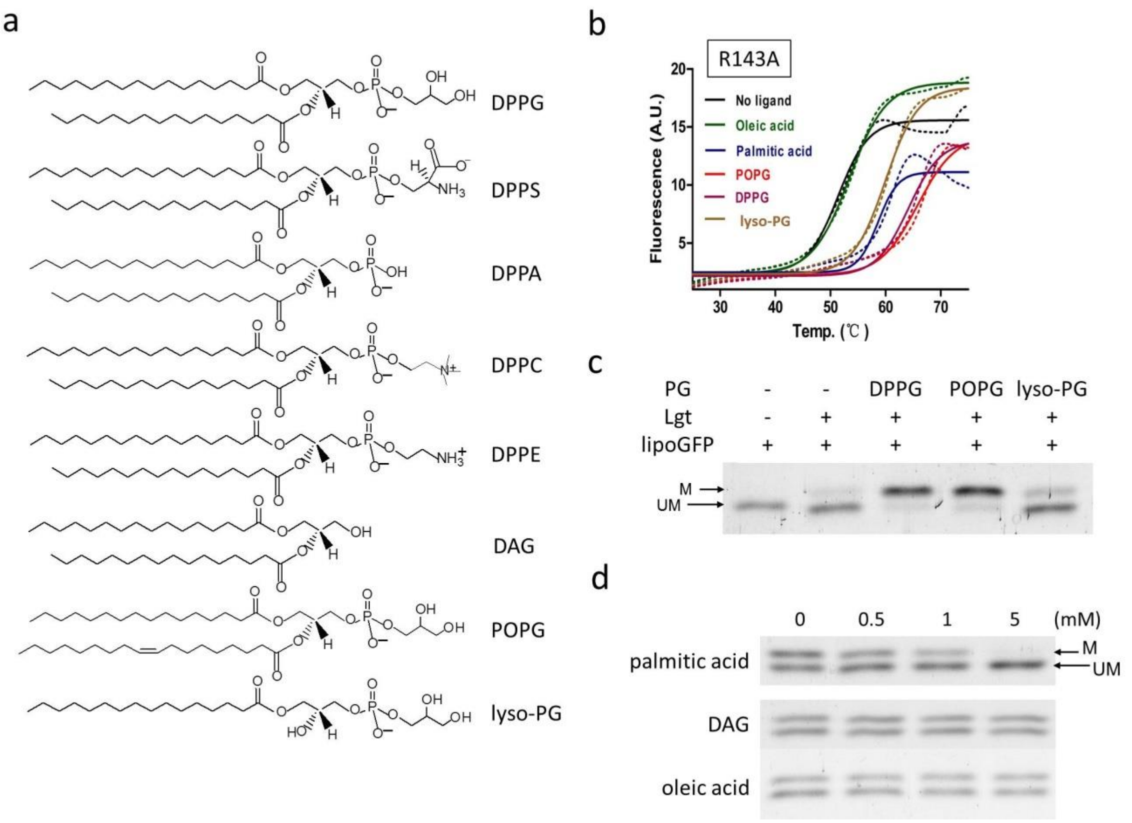

Supplementary Figure 2. Lipid substrate specificity of Lgt using different phospholipids and the in vitro Lgt assay.

a. Chemical structures of phospholipids (except DAG) with either dipalmitoyl tails but varied phosphorous head groups or with oleoyl tail in the sn-2 position as in POPG and only palmitoyl in the sn-1 position (lyso-PG). These lipids were used in the *in vitro* functional and thermofluor assays in this work:

DPPG, 1,2-dihexadecanoyl-sn-glycero-3-phospho-(1'-rac-glycerol);

DPPS, 1,2-dihexadecanoyl-sn-glycero-3-phospho-L-serine);

DPPA, 1,2-dipalmitoyl-sn-glycero-3-phosphate;

DPPC, 1,2-dihexadecanoyl-sn-glycero-3-phosphocholine;

DPPE, 1,2-dihexadecanoyl-sn-glycero-3-phosphoethanolamine;

DAG, diacyl-glycerol (16:0);

lyso-PG, 1-palmitoyl-2-hydroxy- sn-glycero-3-phospho- (1'-rac-glycerol) (16:0/0:0);

POPG, 1-hexadecanoyl-2-(9Z-octadecenoyl)- sn-glycero-3-phospho- (1'-rac-glycerol) (16:0/18:1(9Z)).

_b._ Thermostability of the R143A variant in the presence of different lipids. Thermofluor results of (10 μM) R143A in the presence of 100 μM of either palmitic acid, oleic acid, lyso-PG, DPPG, or POPG are shown. Dash lines represent experimental data, and solid lines represent fitting to a Boltzmann model.

_c._ Lipid-donor specificity of WT Lgt in regarding with varied lipid tails. Conditions of the *in vitro* activity reactions were as following: 50 μl total volume, 10 μM (final) Lgt, 230 μM lipoGFP, and 1 mM specified lipids, at 37°C for 40 min. As expected, DPPG and DOPG were good substrates. Interestingly lyso-PG appeared to be a substrate but a poor one. Uncropped images are shown in Supplementary Fig. 6.

_d._ Inhibition of palmitic acid on Lgt activity. The reaction mixture (WT Lgt, 5 μM; DPPG, 1 mM; lipoGFP, 800 μM; and palmitic acid at the specified concentration) was incubated at 37°C for 12 min, and was then subjected to SDS PAGE and fluorescence imaging. As comparisons, DAG and oleic acid did not shown inhibitory effects. Uncropped images are shown in Supplementary Fig. 6.

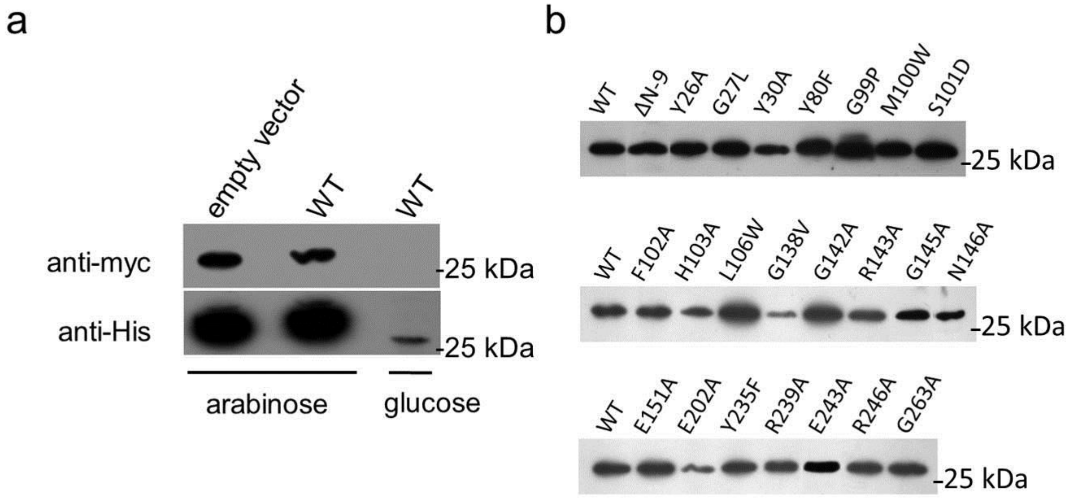

C

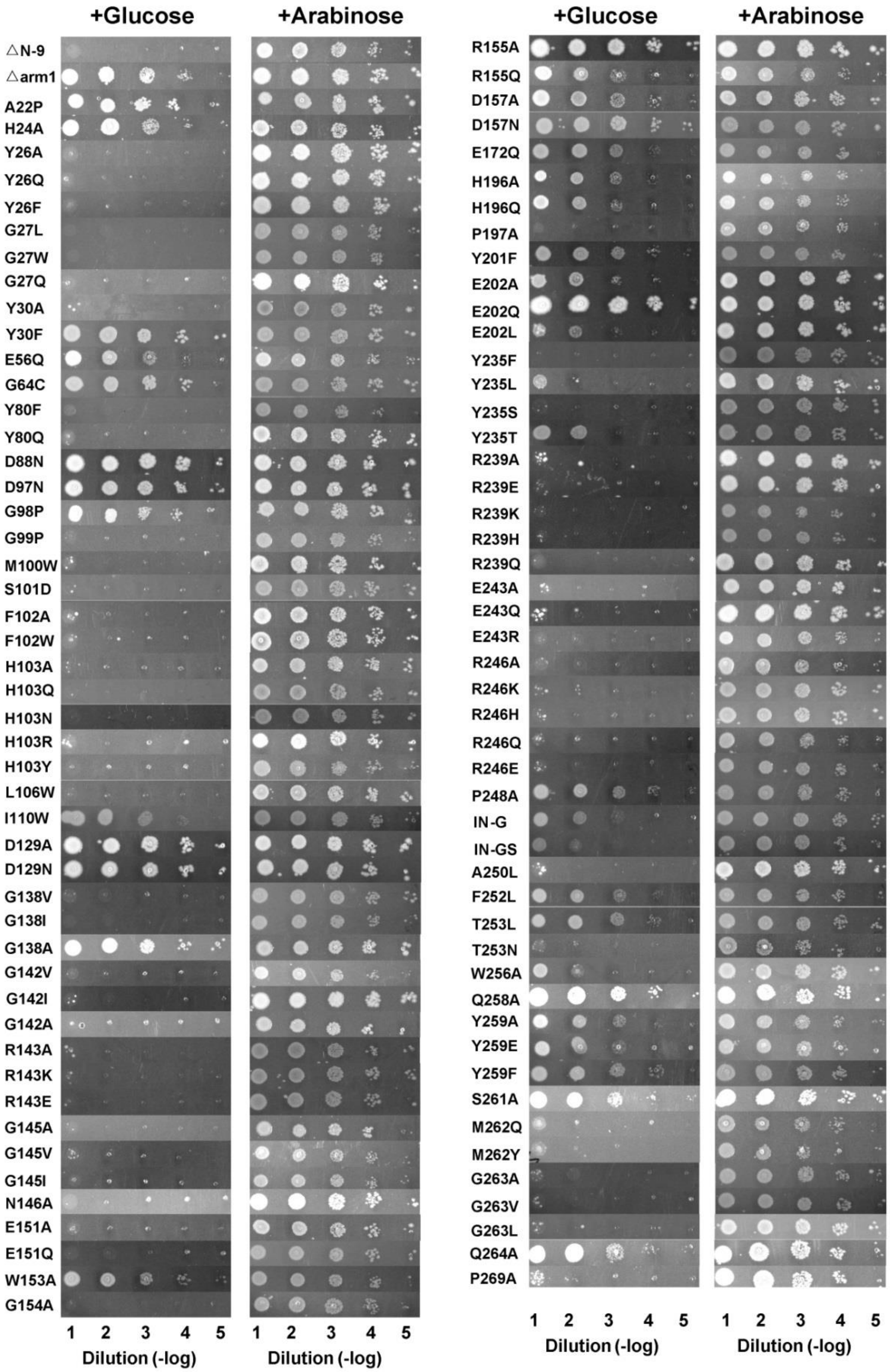
+Glucose
+Arabinose
△N-9
△arm1
A22P
H24A
Y26A
Y26Q
Y26F
G27L
G27W
G27Q
Y30A
Y30F
E56Q
G64C
Y80F
Y80Q
D88N
D97N
G98P
G99P
M100W
S101D
F102A
F102W
H103A
H103Q
H103N
H103R
H103Y
L106W
I110W
D129A
D129N
G138V
G138I
G138A
G142V
G142I
G142A
R143A
R143K
R143E
G145A
G145V
G145I
N146A
E151A
E151Q
W153A
G154A
1 2 3 4 5
Dilution (-log)
1 2 3 4 5
Dilution (-log)
+Glucose
+Arabinose
R155A
R155Q
D157A
D157N
E172Q
H196A
H196Q
P197A
Y201F
E202A
E202Q
E202L
Y235F
Y235L
Y235S
Y235T
R239A
R239E
R239K
R239H
R239Q
E243A
E243Q
E243R
R246A
R246K
R246H
R246Q
R246E
P248A
IN-G
IN-GS
A250L
F252L
T253L
T253N
W256A
Q258A
Y259A
Y259E
Y259F
S261A
M262Q
M262Y
G263A
G263V
G263L
Q264A
P269A
1 2 3 4 5
Dilution (-log)
1 2 3 4 5
Dilution (-log)

Supplementary Figure 3. Complementation assay.

a. Immunoblot of Δ*lgt* strain with and without a complementary plasmid. The rescue plasmid pBAD-Lgt is induced upon addition of arabinose (1 mM), and is repressed following addition of glucose (11 mM). It contains both one myc-tag and one His6-tag. The complementary plasmid (labelled as WT), which contained only His6-tag, showed leaky expression. Leaking expression was sufficient to rescue Δ*lgt* in the presence of glucose (also see Fig. 1b and the Method section). The anti-myc and anti-His antibodies were purchased from CWBIO (Cat. #: CW0082 and CW0299, respectively), and both were 1:2000 diluted. Uncropped images are shown in Supplementary Fig. 6. b. Confirmation of the expression of selected Lgt variants, using anti-His immunoblotting. Uncropped images are shown in Supplementary Fig. 6. c. Results of complementation assays on Lgt variants. For each variant of Lgt, a serial dilution of cell culture was spotted onto solid medium containing either 11 mM glucose (left columns) or 1 mM arabinose (right columns). Photos were taken 16 h after incubation at 37°C.

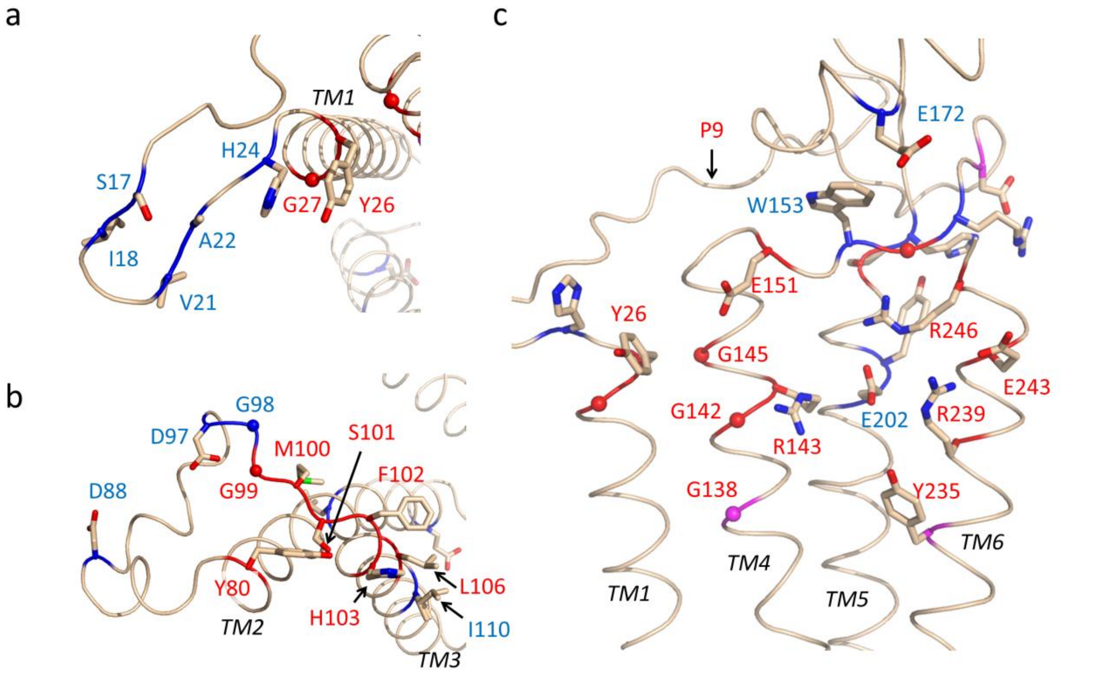

Supplementary Figure 4. More detailed distribution of mutations sites that were included in the *in vivo* screening.

<u>*a.*</u> Arm-1. <u>*b.*</u> The minor TM domain. <u>*c.*</u> The major TM domain. Main chains of residues tolerable to mutations are labeled in blue, those intolerable to mutations are labeled in red, and those in between in magenta.

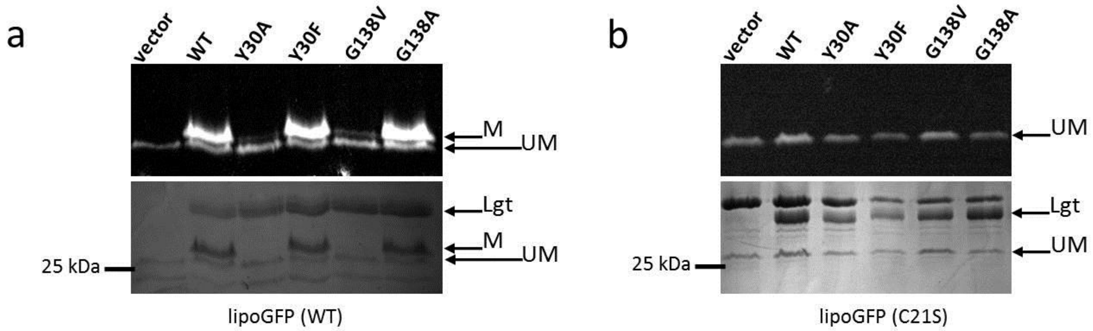

Supplementary Figure 5. ***In vitro*** **affinity assay of selected Lgt variants.**

<u>*a.*</u> The top and bottom panels are images of GFP fluorescence and Coomassie blue-stained gel respectively. WT and other active Lgt variants but not the inactive ones bound lipoGFP and pulled it down effectively as revealed by GFP fluorescence and protein staining. The “vector” lane, which did not contain His-tagged Lgt, served as the background response. The bound form was predominantly the product, lipid-modified lipoGFP, indicating its association with the enzyme even after the reaction. Uncropped images are shown in Supplementary Fig. 6.

<u>*b.*</u> The C21S mutant of lipoGFP did not bind effectively to any tested Lgt variant. Uncropped images are shown in Supplementary Fig. 6.

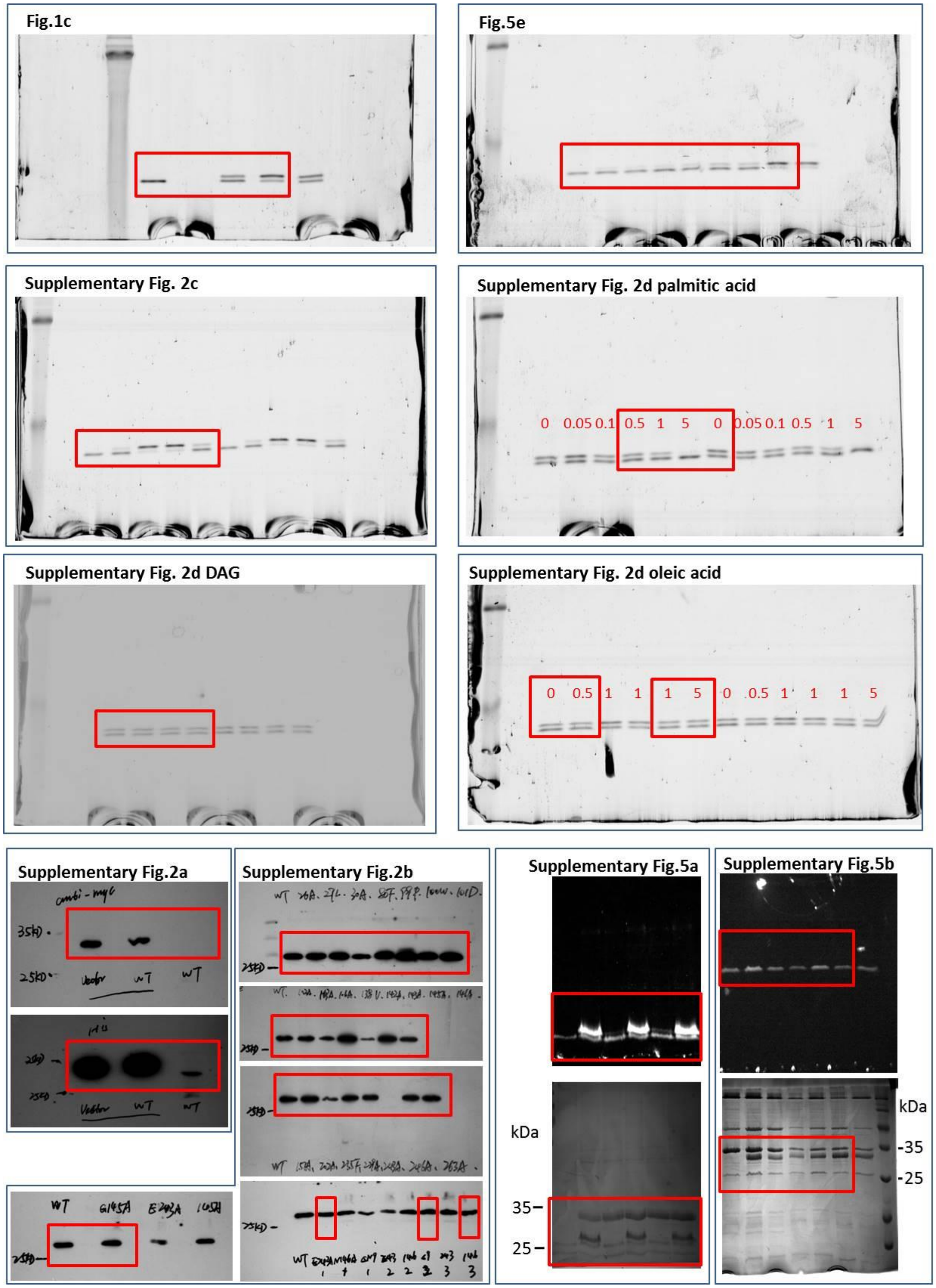

Supplementary Figure 6.Ucropped figures of SDS-PAGE gels and Western Blots.

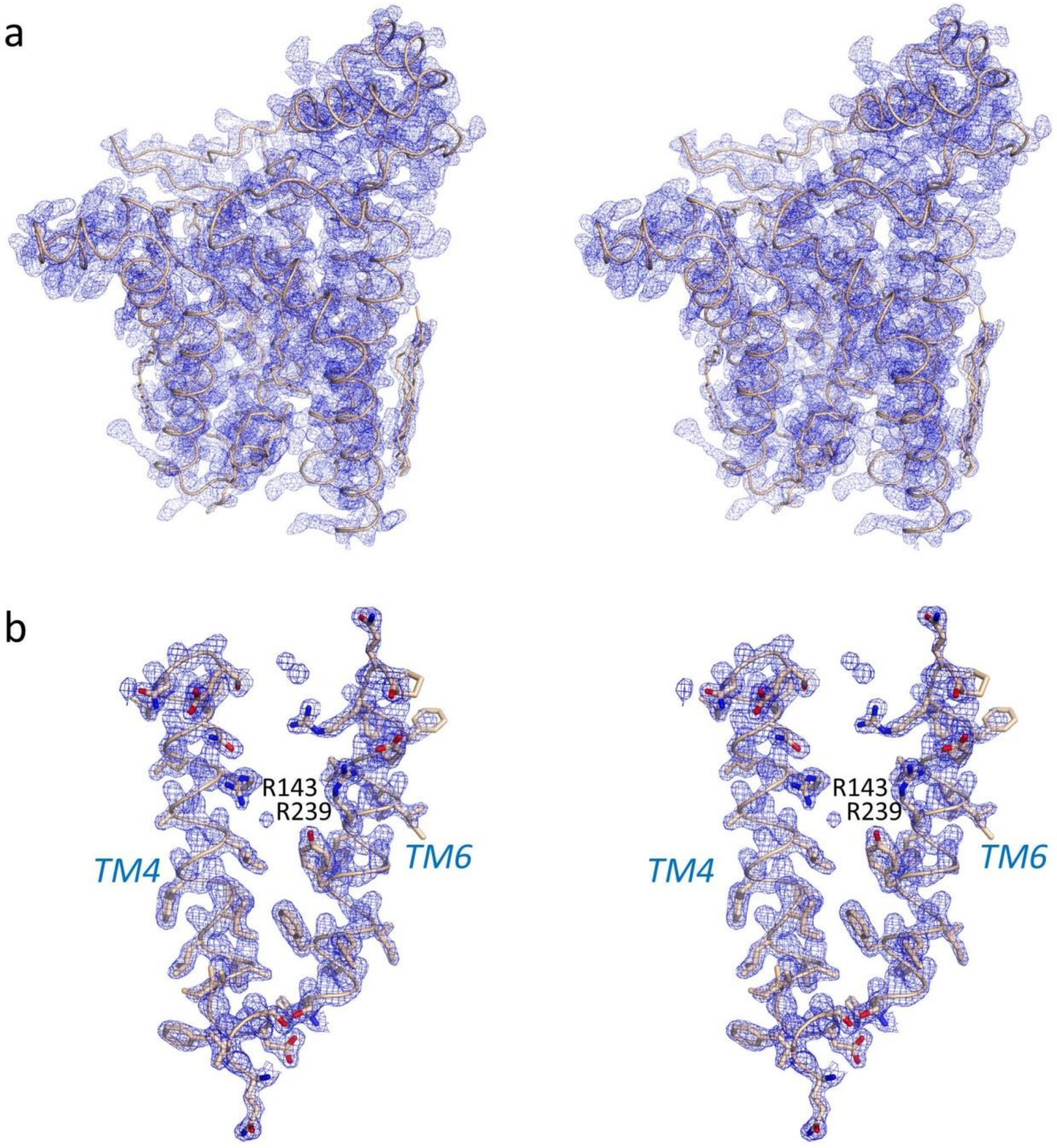

Supplementary Figure 7. Wall-eyed stereo view of 2Fo-Fc electron density map of *Ec*Lgt structure in form-2 at 1.9 Å contoured at 1.0 σ.

a. Overall structure of the *Ec*Lgt

b. Transmembrane helices 4 and 6. Residuals R143 and R239 are labeled.

Supplementary References

1 Schuster-Bockler, B., Schultz, J. & Rahmann, S. HMM Logos for visualization of protein families. *BMC Bioinformatics* **5**, 7 (2004).
